# Pan-cancer analysis combined with experiments explores the oncogenic role of spindle apparatus coiled-coil protein 1 (SPDL1)

**DOI:** 10.1186/s12935-022-02461-w

**Published:** 2022-01-29

**Authors:** Peng Song, Dilinaer Wusiman, Fenglan Li, Xiaoxuan Wu, Lei Guo, Wenbin Li, Shugeng Gao, Jie He

**Affiliations:** 1 Department of Thoracic Surgery, National Cancer Center, National Clinical Research Center for Cancer/Cancer Hospital, Chinese Academy of Medical Sciences and Peking Union Medical College, Beijing, 100021 China; 2 Department of Head and Neck Surgery, National Cancer Center, National Clinical Research Center for Cancer/Cancer Hospital, Chinese Academy of Medical Sciences and Peking Union Medical College, Beijing, 100021 China; 3 Department of Diagnostic Imaging, National Cancer Center, National Clinical Research Center for Cancer/Cancer Hospital, Chinese Academy of Medical Sciences and Peking Union Medical College, 100021 Beijing, China; 4 Department of Pathology, National Cancer Center, National Clinical Research Center for Cancer/Cancer Hospital, Chinese Academy of Medical Sciences and Peking Union Medical College, Beijing, 100021 China

**Keywords:** SPDL1, Pan-cancer, Prognosis, Expression, Genetic alteration, Immune infiltration

## Abstract

**Background:**

The function of spindle apparatus coiled-coil protein 1 (SPDL1) as a cancer-promoting gene has been reported in a number of studies. However, the pan-cancer analysis of SPDL1 is still lacking. Here, we performed this pan-cancer analysis to evaluate the expression and prognostic value of SPDL1 and gain insights into the association between SPDL1 and immune infiltration.

**Methods:**

In this study, based on the datasets of The cancer genome atlas (TCGA), Gene expression omnibus (GEO), The Genotype-Tissue Expression (GTEx) and Clinical Proteomic Tumor Analysis Consortium (CPTAC), we used R4.1.0 software and the online tools, including TIMER2.0, GEPIA2, cBioPortal, Modbase, UALCAN, MEXPRESS, STRING, Ensembl, NCBI, HPA, Oncomine, PhosphoNET and the Kaplan-Meier plotter, to explore the potential oncogenic roles of SPDL1. The expression of SPDL1 was also further verified by immunohistochemistry (IHC) in lung adenocarcinoma (LUAD) tissues.

**Results:**

SPDL1 was overexpressed in most tumors compared with adjacent normal tissues, and SPDL1 expression was significantly correlated with the prognosis in most tumor types. The main type of genetic mutation of SPDL1 was missense mutation and the frequency of R318Q/W mutation was highest (4/119). The expression of SPDL1 was closely associated with genomic instability. The SPDL1 phosphorylation levels in S555 was enhanced in ovarian cancer. The SPDL1 expression was positively correlated with the immune infiltration of CD8+ T-cells and cancer-associated fibroblasts (CAFs) in most of the tumor types. Nuclear division, organelle fission and chromosome segregation were involved in the functional mechanisms of SPDL1.

**Conclusions:**

These findings suggested that SPDL1 might serve as a biomarker for poor prognosis and immune infiltration in cancers, shedding new light on therapeutics of cancers.

**Supplementary Information:**

The online version contains supplementary material available at 10.1186/s12935-022-02461-w.

## Introduction

SPDL1 is also called coiled-coil domain containing-99 (CCDC99), which encodes Spindly. Spindly is a coiled-coil domain-containing protein that involves in cell division and chromosome segregation, including mitotic spindle formation and spindle checkpoint [1, 2]. The spindle assembly checkpoint (SAC) monitors the attachment between microtubules and kinetochores during prometaphase. In response to unattached kinetochores, the SAC generates the mitotic checkpoint complex (MCC), a multimeric assembly that can delay chromosome segregation [3, 4]. Spindly involves in the SAC silencing which causes the inhibition of MCC formation, subsequently changes the metaphase status to anaphase status [5–7]. The inhibition of spindly leads to unstable interactions between centromeres and microtubules, leading to defects in chromosomal arrangement, prophase delay, and cell accumulation during mitosis [8, 9]. Besides, Spindly can interact with components of the RZZ complex (Rod/ZW10/Zwilch) and the dynein-dynactin complex at kinetochores [9–11]. Spindly colocalizes with dynein/dynactin at the leading edge of migrating cells and cells lacking Spindly migrate slower than wild type cells, which means Spindly may play an important role in the cell migration process [12, 13].

In addition to its role in normal tissue cells, SPDL1 is also involved in the tumor growth and prognosis. To date, several studies have reported that the CCDC99 over-expressed in tumor tissues in different cancer types, including lung cancer [14], oral squamous cell carcinoma (OSCC) [15], pancreatic ductal adenocarcinoma (PDAC) [16]. High expression of Spindly was an independent indicator for poor prognosis and associated with cellular proliferation in OSCC [15]. However, the recent study showed that overexpression of SPDL1 in PDAC was associated with a better prognosis [17]. SPDL1 was defined as a candidate tumor suppressor in colorectal cancer (CRC) to play an essential role in the downstream of Myocardin-related transcription factor B (MRTFB) that could regulate CRC growth and survival [18]. Moreover, Spindly inhibition could enhance the cytotoxic activity of paclitaxel due to an increase in the duration of mitotic delay which provide a novel insight into circumventing paclitaxel resistance in cancer treatment [19]. Interestingly, the variant of SPDL1 reduce the incidence of different cancer types due to lower chromosomal alterations accumulated over time [20]. SPDL1 was identified as tumor infiltration CD8+ T cell-related genes in a prognostic model in LUAD, which indicate the potential relation of SPDL1 and immunity [21].

Considering the heterogeneity of different tumor types, a pan-cancer analysis is needed to get a comprehensive understanding of SPDL1. In this study, we dissected the oncogenic role of SPDL1 across all cancer types of TCGA from various aspects, including gene expression, genetic alteration, methylation level, protein phosphorylation, immunology, survival prognosis, and gene enrichment analysis, which can further uncover the potential molecular mechanism of SPDL1 in cancers and its value in clinical prognosis of cancer patients.

## Materials and methods

### Gene expression analysis

We observed the differential expression of SPDL1 between tumor and normal tissues adjacent to carcinoma in the different tumors or specific tumor subtypes of the TCGA project by the “Gene_DE” of TIMER2.0 (Tumor immune estimation resource, version 2) website (http://timer.cistrome.org/). For some tumors without normal tissues [e.g., DLBC (Lymphoid Neoplasm Diffuse Large B-cell Lymphoma), LGG (Brain Lower Grade Glioma), etc.], we obtained box plots of the expression difference between the tumor tissues and the corresponding normal tissues of the GTEx (Genotype-tissue expression) database, using the “Expression analysis-Box Plots” module of the GEPIA2 (Gene expression profiling interactive analysis, version 2) web server (http://gepia2.cancer-pku.cn/#analysis), assuming that the setting cut-off value are P-value cutoff = 0.01, log2FC (Fold change) cutoff =1, and “Match TCGA normal and GTEx data” [22]. In addition, through the “Pathological Stage Plot” module of GEPIA2, we obtained violin plots of the SPDL1 expression in different pathological stages (I, II, III, and IV) of all TCGA tumors. Other related gene expression analyses were detailed in the Additional file 1.

### Survival prognosis analysis

We used the “Survival Map” module of GEPIA2 to obtain the overall survival (OS) and disease-free survival (DFS) significance map data of SPDL1 across all TCGA tumors. Group cutoff was set as median to separate the samples into two groups. The statistical difference between the curves was measured by the log-rank test and we also obtained the survival plots through the “Survival Analysis” of GEPIA2. The detailed information of survival prognosis analysis in Kaplan-Meier plotter were displayed in Additional file 1.

### Immunohistochemistry

For SPDL1 expression analysis in tumor tissues, we obtained archival formalin-fixed paraffin-embedded (FFPE) specimens of 158 LUAD from Cancer Hospital, Chinese Academy of Medical Science, Beijing, China. Immunohistochemistry (IHC) were performed on the tissue microarray chips according to the protocol of a previous study [23], with anti-SPDL1 antibody (1:100, NBP2-47517, NOVUS). This study was approved by our institutional review board of Cancer Hospital, Chinese Academy of Medical Science.

The score was determined by multiplying the score of staining intensity and score of positive cells. The score of staining intensity was scored as follows: 0 for no staining, 1 for weak staining, 2 for moderate staining and, 3 for strong staining. The score for percentage of positive area was kept in the range of 1 to 4, based on: less than 5% positive cells for 0; 5–25% positive cells for 1; 26–50% positive cells for 2; 51–75% positive cells for 3; greater than 75% positive cells for 4. Further, for statistical analyses the scores of 0–6 were treated as low expression, scores of 7–12 as high expression. OS of high- and low-SPDL1 subgroups of patients was compared using the Kaplan–Meier method with the log-rank test. The data in this part were analyzed and plotted by using the “survival” package of R4.1.0 software (https://www.r-project.org/).

### Genetic alteration analysis

We logged into the cBioPortal website (https://www.cbioportal.org/), and chose the “TCGA Pan Cancer Atlas Studies” in the “Quick select” section and clicked the “Query By Gene” button. After entering “SPDL1”,we can query of the genetic alteration characteristics of SPDL1. The results of the mutation type, CNA (Copy number alteration) and alteration frequency across 10,967 samples in 32 studies were observed in the “Cancer Types Summary” module. We downloaded SPDL1 protein structure in website Modbase [24] (https://modbase.compbio.ucsf.edu/). The 3D (Three-dimensional) structure of the mutated site information of SPDL1 were displayed by the Swiss-Pdbviewer 4.1.0 software (https://spdbv.vital-it.ch/disclaim.html) via choosing “act on ribbon” and “Secondary Structure Succession”. We also obtained the data on the overall, disease-free, disease-specific, and progression free survival differences for specific cancer types with or without SPDL1 genetic alteration in the “Comparison” module. Kaplan-Meier plots can be obtained with log-rank P-value.

### Immune infiltration analysis

We used the “Immune” module of the TIMER2.0 which provides more robust estimation of immune infiltration levels using six state-of-the-art algorithms, to investigate the association between immune infiltrates and SPDL1 expression in TCGA tumors [25]. The cancer-associated fibroblasts (CAFs) and immune cells of CD8+ T-cells were selected. We applied the EPIC, MCPCOUNTER, CIBERSORT-ABS, QUANTISEQ and TIDE algorithms for immune infiltration estimations. We obtained a heatmap table of the Spearman’s correlations between SPDL1 expression and the abundance of the immune cell type. Scatter plots show the correlation of SPDL1 expression with tumor purity (left) and with the infiltration level (right).

### Association between SPDL1 expression and genomic instability

We curated a list of genomic instability scores from a previous study, which was composed of tumor mutation burden (TMB), number of neoantigens, microsatellite instability (MSI), the aneuploidy, loss of heterozygosity (LOH) and the homologous recombination deficiency (HRD) score [26]. The correlation analysis was performed by the Spearman’s method, using the “cor.test” package of R4.1.0 software. It was visualized by a radar map, designed by the R-package “fmsb”.

### SPDL1-related gene enrichment analysis

The Protein–protein interaction (PPI) network was predicted using Search Tool for the Retrieval of Interacting Genes (STRING; https://string-db.org/) (version 11.5) online database [27]. We input “SPDL1” and “Homo sapiens” in the “protein by name” module. In the present study, PPI network of DEGs was constructed using STRING database, and an interaction with a combined score >0.150 was considered statistically significant. In our study, we analyze 50 interactors. Visualization was carried out using the Cytoscape3.8.2 software (http://www.cytoscape.org/), an open-source bioinformatics software platform for visualizing molecular interaction networks [28].

“Similar Gene Detection” button of GEPIA2 were used to search for the top 100 genes that has a similar expression pattern with SPDL1 in different cancer types including TCGA tumor tissues and normal tissues. The “correlation analysis” module was applied to compute the correlation of SPDL1 and some selected genes. It was indicated the P-value and the correlation coefficient (R). Subsequently, we logged into TIMER2.0 and applied the “Gene_Corr” module to get the heatmap data with selecting the ‘Purity Adjustment’ checking box of the selected genes, which were used to analysis the correlation in the previous step. It supplied the purity-adjusted partial spearman’s rho value as the degree of their correlation.

We applied Venn Diagrams in Bioinformatics & Evolutionary Genomics website (http://bioinformatics.psb.ugent.be/webtools/Venn/), which were used to calculate and draw custom Venn diagrams comparing the SPDL1-binding and interacted genes. The combined two sets of data were used to conduct Kyoto encyclopedia of genes and genomes (KEGG) pathway and Gene ontology (GO) enrichment analysis. We applied the “Bioconductor-cluster Profiler” (http://www.bioconductor.org/packages/release/bioc/html/clusterProfiler.html) R4.1.0 package to conduct analysis. GO term analysis was classified into three subgroups, namely biological process (BP), cellular component (CC) and molecular function (MF). Bubble charts were plotted by using the ggplot2 (https://ggplot2.tidyverse.org/) package of R4.1.0 software. P < .05 was considered a threshold.

### Gene set enrichment analysis

In addition, Gene set enrichment analysis (GSEA) was used to inspect the statistical significance of a defined sets of genes and verify the differences between two biological states [29]. We divided samples of LUAD and LUSC in TCGA into two subgroups on the grounds of the median expression level of SPDL1 respectively. GO gene sets were analyzed by the “clusterProfiler” and “enrichplot” R package to identify functional terms and pathways. Gene set permutations were executed 100 times for each analysis. The criteria of significantly enriched pathways were normalized P < .05.

## Results

### Gene expression analysis

In this study, we aimed to explore the oncogenic role of human SPDL1 (NM_017785.5 for mRNA or NP_060255 for protein, Additional file 2: Fig. S1A). The SPDL1 protein structure was conserved among different species (e.g., H. sapiens, *C. lupus*, *B. taurus*, *G. gallus*, etc.) (Additional file 2: Fig. S1B). The evolutionary relationship of SPDL1 protein among different species were shown by the plot of the phylogenetic tree in Additional file 2: Fig. S2.

We calculated the expression of SPDL1 in different tissue types and blood cell types. Enhanced RNA tissue specificity was shown in the normal testis samples, according to the combined data from The Human protein atlas (HPA), The Genotype-Tissue Expression (GTEx) and Functional Annotation of Mammalian Genomes 5 (FANTOM5) (Additional file 2: Fig. S3A). It also exhibited the elevated SPDL1 expression in thymus and spinal cord (Additional file 2: Fig. S3A). Additional file 2: Fig. S3B displayed the low RNA blood cell type specificity when analyzing the SPDL1 expression in consensus dataset (HPA, Monaco, Schmiedel) .

**Fig. 1:**
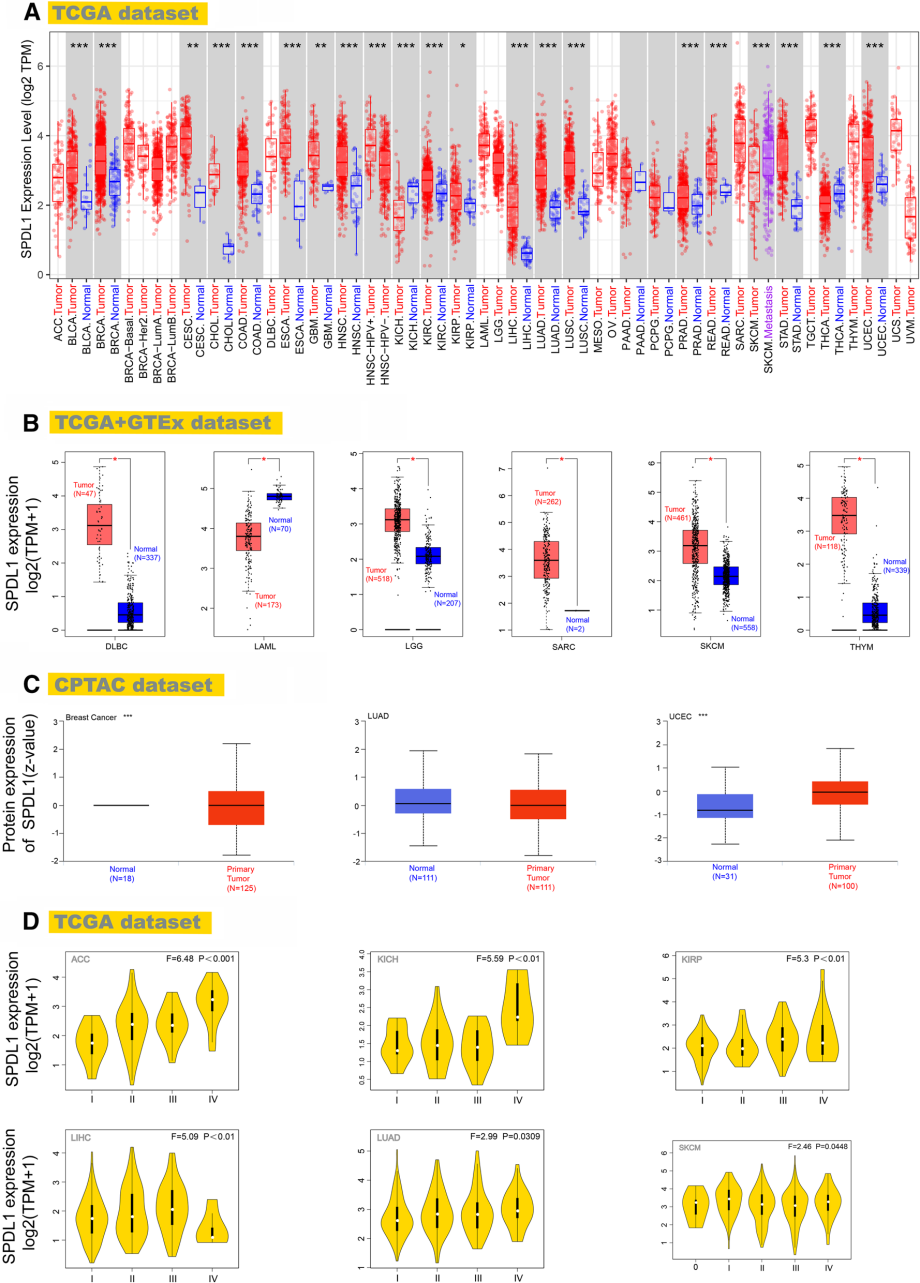
SPDL1 expression in different cancer types. **A** The expression of the SPDL1 gene in different tumors or specific cancer subtypes. *P < .05; **P < .01; ***P < .001. **B** The normal tissues of the GTEx database were included for the analysis of SPDL1 expression in DLBC, LAML, LGG, SARC, SKCM, THYM with the TCGA project. **P < .01. **C** SPDL1 protein expression level in the primary tumor tissues and normal tissues of breast cancer, LUAD and UCEC (P < .001) from the CPTAC dataset. ***P < .001. **D** SPDL1 gene expression level analyzed by the pathological stages (stage I, stage II, stage III, and stage IV) of ACC, KICH, KIRP, LIHC, LUAD, SKCM

The TIMER2.0 was used to analyze the expression of SPDL1 in all cancer types. SPDL1 expression in the tumor tissues of Bladder Urothelial Carcinoma (BLCA), Breast invasive carcinoma (BRCA), Cholangiocarcinoma (CHOL), Colon adenocarcinoma (COAD), Esophageal carcinoma (ESCA), Head and Neck squamous cell carcinoma (HNSC), Kidney chromophobe (KICH), Kidney renal clear cell carcinoma (KIRC), Liver hepatocellular carcinoma (LIHC), LUAD, Lung squamous cell carcinoma (LUSC), Prostate adenocarcinoma (PRAD), Rectum adenocarcinoma (READ), Stomach adenocarcinoma (STAD), Thyroid carcinoma (THCA), Uterine Corpus Endometrial Carcinoma (UCEC), Cervical squamous cell carcinoma and endocervical adenocarcinoma (CESC), Glioblastoma multiforme (GBM) and Kidney renal papillary cell carcinoma (KIRP) (P < .05) was higher than that in the corresponding tumor adjacent tissues (Fig. 1A). Interestingly, SPDL1 expression in HPV positive HNSC was higher than HPV negative HNSC (P < .001) (Fig. 1A).

Due to insufficient normal samples for some TCGA tumors, we added normal tissues from the GTEx data collection. We assessed the expression of SPDL1 between the normal tissues and tumor tissues of DLBC, Acute Myeloid Leukemia (LAML), LGG, Sarcoma (SARC), Skin Cutaneous Melanoma (SKCM), Thymoma (THYM) (P < .01) (Fig. 1B). However, as shown in Additional file 2: Fig. S4A, there was no significant difference in other tumors, including Adrenocortical carcinoma (ACC), Ovarian serous cystadenocarcinoma (OV), Testicular Germ Cell Tumors (TGCT), Uterine Carcinosarcoma (UCS).

Protein expression of SPDL1 was higher in the primary tumor tissues than normal tissues of breast cancer, LUAD and UCEC (P < .001) in the CPTAC dataset (Fig. 1C). The comparative analysis of several studies in the Oncomine database also showed that SPDL1 was highly expressed in cervical cancer, colorectal cancer, kidney cancer, head and neck cancer, lung cancer and sarcoma compared with normal tissues (P < .001) (Additional file 2: Fig. S5). In several cancers, SPDL1 expression was found to be significantly correlated with the pathological stages by the “Pathological Stage Plot” module of GEPIA2 in ACC, KICH, KIRP, LIHC, LUAD, SKCM (P < .05) (Fig. 1D), but not in others (Additional file 2: Fig. S4B).

### Survival analysis

**Fig. 2:**
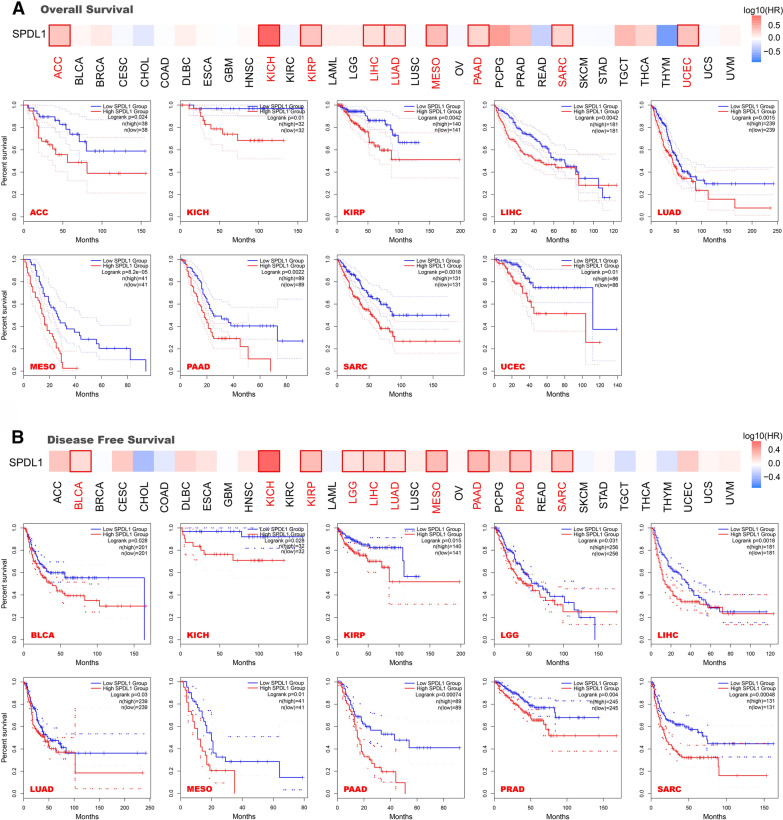
Correlation between SPDL1 gene expression and the prognosis of different tumors in TCGA. **A** Overall survival. **B** Disease-free survival

High-expression groups and low-expression groups were classified by the median cut-off value of their expression levels to obtain the correlation between gene expression and prognosis of different tumors. Survival analysis showed that higher expression was associated with poorer OS in ACC, KICH, KIRP, LIHC, LUAD, MESO, PAAD, SAC, UCEC (P < .05) (Fig. 2A). As shown in Fig. 2B, high SPDL1 expression in BLCA, KICH, KIRP, LGG, LIHC, LUAD, MESO, PAAD, PRAD, SARC was linked to poorer DFS (P < .05).

In addition, the Kaplan–Meier plotter tool was used to perform survival prognosis analysis based on the different GEO datasets, including breast, lung, ovarian, gastric, and liver cancers. The results revealed that high SPDL1 expression was correlated with the poor OS, distant metastasis-free survival (DMFS), progress-free survival (PFS) and relapse-free survival (RFS) in liver cancer (P < .001) (Additional file 2: Fig. S6A). Additionally, high levels of expression were linked to poor OS (P = .0011), DMFS (P < .001), post-progression survival (PPS) (P < .001) and RFS (P < .001) in breast cancer (Additional file 2: Fig. S6B). In contrast, lowly expressed SPDL1 was related to poor OS (P < .001), first progression (FP) (P < .05) and PPS (P < .001) in gastric cancer (Additional file 2: Fig. S6C). However, the correlation between SPDL1 and prognosis in lung cancer was more complicated. A high expression level of SPDL1 was associated with poor OS (P < .05) and FP (P < .01), however, a low expression level of SPDL1 was related to poor PPS (P < .001) (Additional file 2: Fig. S6D). In ovarian cancer, a high expression level was associated with poor OS (P < .001), PFS (P < .001), PPS (P = .045) (Additional file 2: Fig. S6E). The forest figure showed the correlation between expression and prognosis more clearly in all cancer types (P < .05) (Additional file 2: Fig. S7). We also performed the subgroup analysis using selected clinical factors in the Kaplan-Meier plotter tool and obtained different results (Additional file 3: Tables S1–S5). It suggested that the prognosis in high and low SPDL1 expression was different in different subgroups. The above findings showed that high SPDL1 expression was correlated with inferior prognosis in most cancer types.

### Validation of the SPDL1 expression in LUAD

**Fig. 3:**
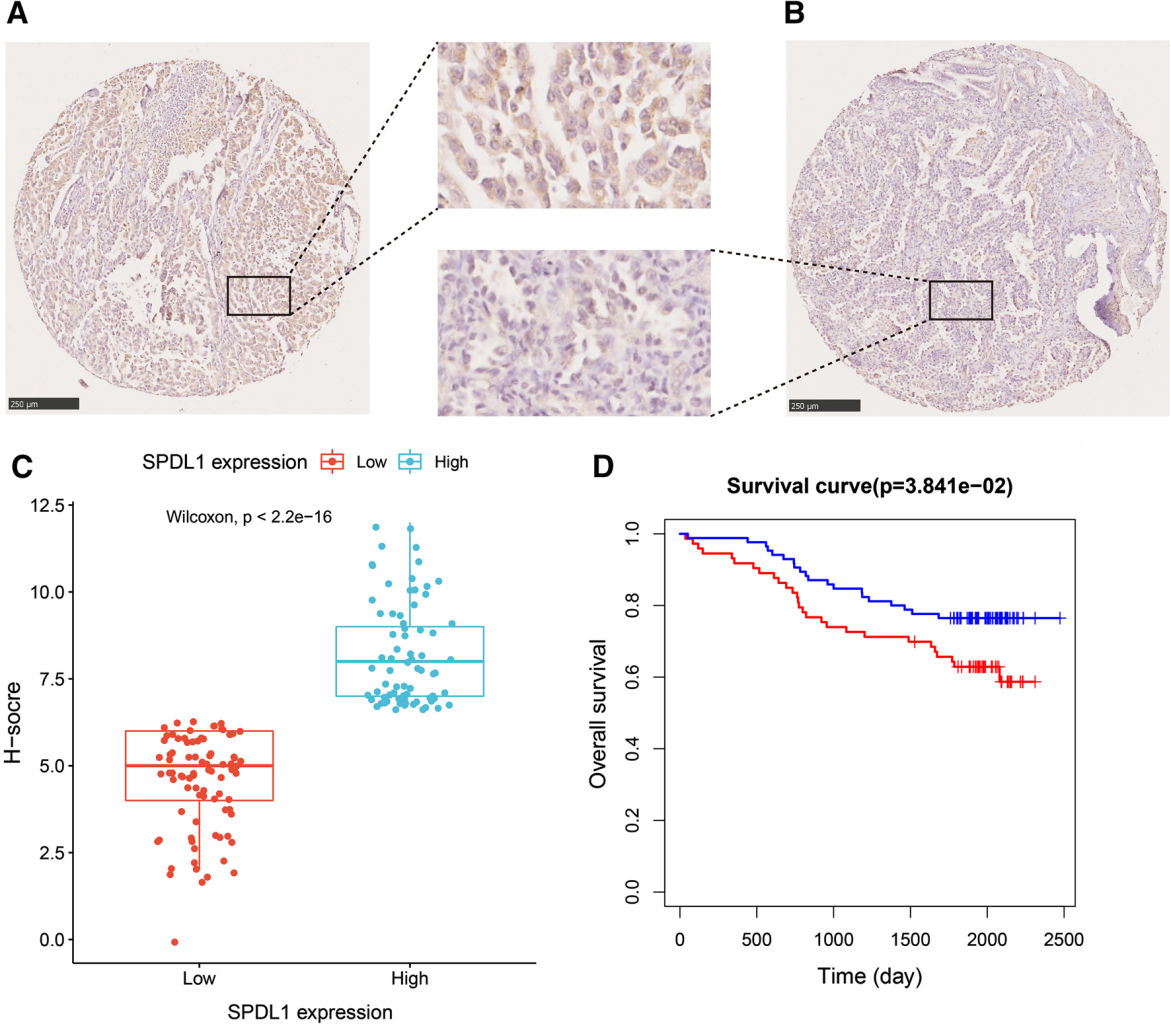
Validation of the expression of SPDL1 in LUAD. IHC staining of SPDL1 in **A** high-expression and **B** low-expression of SPDL1 in the tissue microarray. **C** Differences in SPDL1 expression scores between two groups of the tissue microarray were presented as a boxplot. **D** Kaplan–Meier plots of the OS in the high-SPDL1 and low-SPDL1 expressed subgroups of the tissue microarray cohort.

In order to verify the above result that high SPDL1 expression was significantly correlated with poor prognosis in LUAD, we further performed immunohistochemical staining of the SPDL1 in a tissue microarray (158 samples) (Fig. 3A, B). The distribution of SPDL1 expression in two groups was visualized in a boxplot (Fig. 3C). The result was consistent with the previous result that high SPDL1 expression was significantly correlated with poor OS in LUAD (P = .03841) (Fig. 3D).

### Genetic alteration analysis

**Fig. 4:**
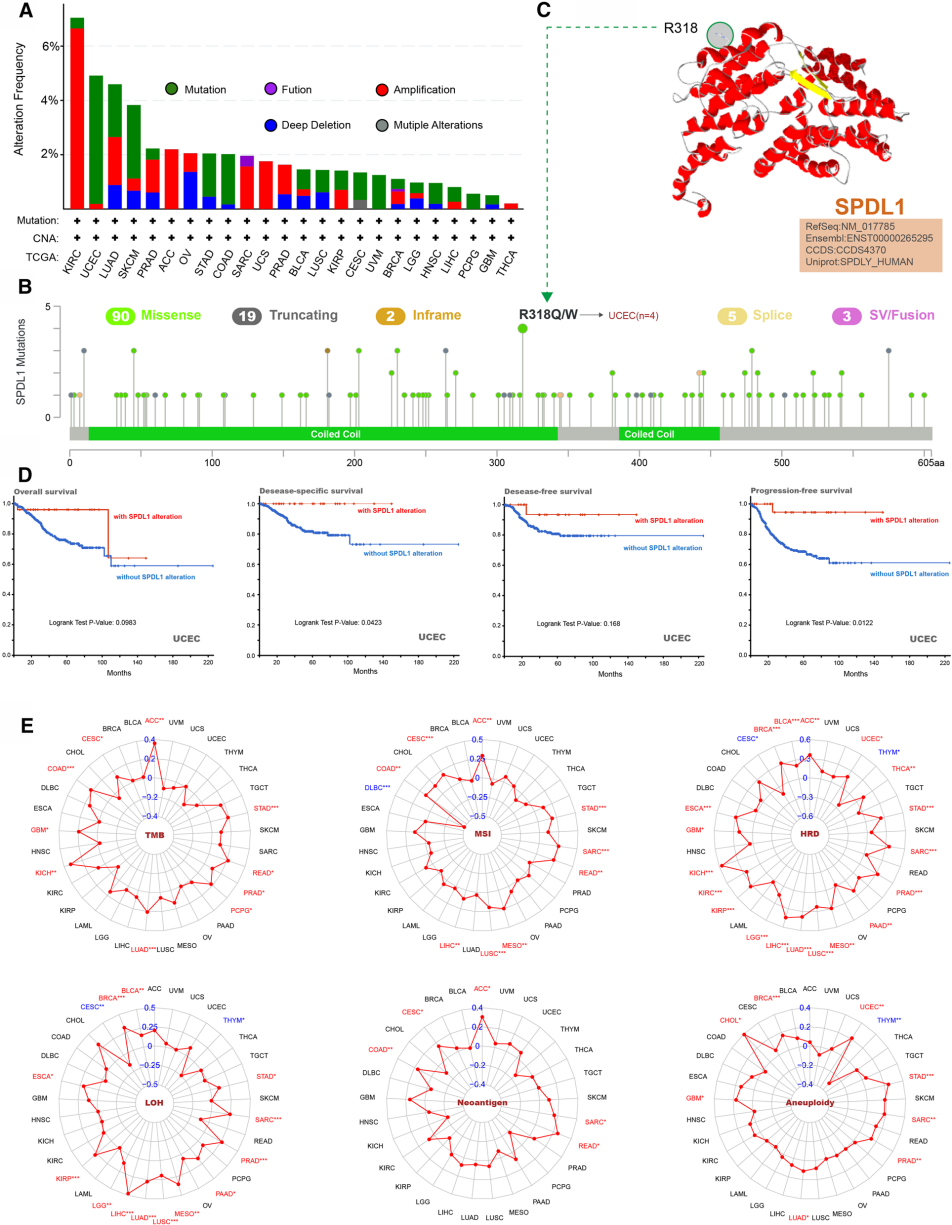
SPDL1 mutation in different cancer types of TCGA. **A** The alteration frequency with mutation type was displayed. **B** The mutation sites were displayed. **C**. The mutation site of R318 in the 3D structure of SPDL1 were displayed. **D** The potential correlation between mutation status and OS, DSS, DFS, and PFS of UCEC were analyzed by the cBioPortal tool. **E** Relationships between SPDL1 expression and genomic instability, including TMB, MSI, HRD, LOH, number of neoantigens and the aneuploidy. OS: overall survival. DSS: disease-specific survival. DFS: disease-free survival. PFS: progress-free survival. TMB: tumor mutational burden; MSI: microsatellite instability; HRD: homologous recombination deficiency; LOH: loss of heterozygosity. *p < .05, **p < .01, and ***p < .001

The genetic alteration status of SPDL1 was investigated in different tumor types of the TCGA. Patients with KIRC had the highest alteration frequency of SPDL1 (7.05%, 36 cases) and “amplification” accounts for the most majority of all alterations (6.65%, 34 cases) (Fig. 4A). Patients with UCEC had the second alteration frequency (4.91%, 26 cases) and the “mutation” type was the primary type (4.73%, 25 cases). The distribution of the SPDL1 genetic mutation were shown in Fig. 4B. The main type of genetic mutation of SPDL1 was missense mutation and the frequency of R318Q/W mutation was highest (4/119), which was able to induce a missense mutation of the SPDL1 gene and translation from R (Arginine) to Q (Glutamine) /W (Tryptophan) at the number of 318 site of SPDL1 protein (Fig. 4B). The R318 site was also displayed in the 3D structure of SPDL1 protein (Fig. 4C). In addition, we explored the potential association between SPDL1 gene alteration and clinical survival in patients with different cancer types. As shown in Fig. 4D, compared with UCEC cases without SPDL1 alteration, cases with SPDL1 alteration showed better prognosis in disease-specific survival (DSS) (P = .0423) and PFS (P = .0122), but not in DFS (P = .168) and OS (P = .0983).

### Association of SPDL1 expression with genomic instability

The dysregulation of the SPDL1 expression may lead to chromosomal arrangement, prophase delay, and cell accumulation during mitosis, which in turn may promote genome instability. We thus assessed the relationship between SPDL1 expression and genome integrity. We used a list of scores that measure the signatures of genome instability, including TMB, HRD, LOH, number of neoantigens and the aneuploidy. Our results indicated that TMB was positively correlated with the SPDL1 expression in ten types of cancer, including ACC, STAD, READ, PRAD, PCPG, LUAD, KICH, GBM, COAD and CESC (Fig. 4E). The results showed that SPDL1 expression was positively associated with MSI among the 8 tumors, including ACC, STAD, SARC, READ, MESO, LUSC, LIHC, COAD and CESC, and negatively associated with MSI in in DLBC (Fig. 4E).

Our results also indicated that SPDL1 expression was significantly correlated with HRD, LOH, number of neoantigens, the aneuploidy in 21, 15, 5 and 9 cancer types respectively. Among these, SPDL1 expression had a positive correlation with HRD in 19 cancer types (ACC, BLCA, BRCA, ESCA, GBM, KICH, KIRC, KIRP, LGG, LIHC, LUAD, LUSC, MESO, PAAD, PRAD, SARC, STAD, THCA, UCEC) (Fig. 4E), and a negative correlation with HRD in CESC and THYM (Fig. 4E). In 5 cancer types, including ACC, CESC, COAD, READ, and SARC, SPDL1 expression was positively correlated with the aneuploidy (Fig. 4E). Our results showed that SPDL1 expression was significantly correlated with the aneuploidy in 9 cancer types (BRCA, CHOL, GBM, LUAD, PRAD, SARC, STAD, THYM, UCEC) (Fig. 4E).

### DNA methylation analysis

DNA methylation plays a key role in the early development and process of cancer, and the pattern of tumor methylation is different from that of normal tissues [30–32]. Therefore, we analyzed the SPDL1 methylation levels in cancer tissues and normal tissues using the UALCAN. The methylation degrees of the SPDL1 promoter in tumor tissues were higher than that in normal tissues in BLCA, COAD, CESC and ESCA (Additional file 2: Fig. S8A). In contrast, the methylation levels of SPDL1 in HNSC, KIRC, KIRP, SARC, TGCT and UCEC tumor tissues were lower than that in normal tissues (Additional file 2: Fig. S8B). Next, we used MEXPRESS to access the possible relationship between SPDL1 methylation and expression of tumors in TCGA. Because of an interest in the lung cancer cases, we investigated the correlation between SPDL1 methylation and expression in LUAD and LUSC. We observed that the methylation of SPDL1 was correlated with gene expression in the regions, such as cg05722931 (P < .01, R = −  .130) in LUAD, cg13043177 (P < .05, R = .102) and cg11437374 (P < .01, R = .155) in LUSC (Additional file 2: Fig. S8C, D).

### Protein phosphorylation analysis

The comparison of the SPDL1 phosphorylation levels between normal tissues and primary tumor tissues was analyzed in breast cancer and ovarian cancer with the CPTAC dataset. The SPDL1 phosphorylation sites in ovarian cancer were shown in Additional file 2: Fig. S9A, and only the comparison of SPDL1 phosphorylation levels in S555 with ovarian cancer showed the significant differences (Additional file 2: Fig. S9B). We used the PhosphoNET database to analyze phosphorylation of SPDL1 and the detail information of S555 phosphorylation were displayed in Additional file 2: Fig S9C.

### Immune cell infiltration analysis

**Fig. 5:**
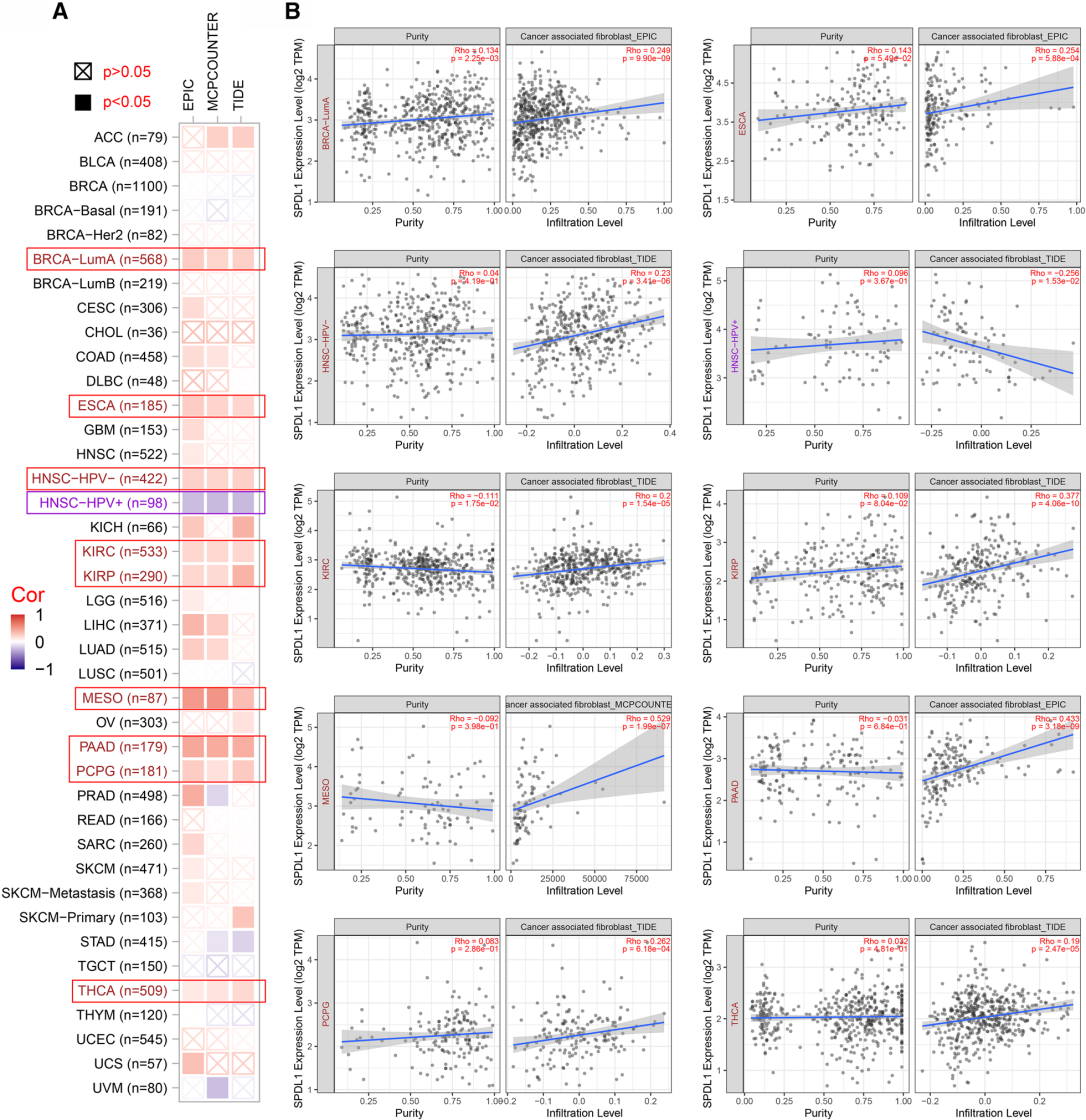
Correlation between the SPDL1 expression and cancer associated fibroblast in all cancer types of TCGA. **A** Different algorithms (EPIC, MCPCOUNTER, TIDE) were used to explore the correlation between the infiltration level of CAFs and the SPDL1 gene expression in all cancer types of TCGA. **B** The scatterplot data of the selected algorithm with the most statistically significant results

Tumor-infiltrating immune cells play an important role in tumor control and cancer treatment efficacy [33, 34]. TIMER2.0 integrates six state-of-the-art algorithms, including TIMER, xCell, MCP-counter, CIBERSORT, EPIC and quanTIseq, for immune infiltration estimation. CD8+ T-cells are important effectors in the elimination of human malignancy and play a critical role in tumor immunity and anti-viral immune responses [35]. The correlation between the immune infiltration of CD8+ T-cells and SPDL1 expression in tumor tissues (BRCA, HNSC-HPV+, LIHC, LUSC, PAAD, PRAD, SKCM, THYM, UCEC and UVM) was found to be statistically significant based on four algorithms (Additional file 2: Fig. S10). CAFs, a central element of the TME, are able to influence other components of the TME such as the immune infiltrate [36, 37]. In addition, the correlation between SPDL1 expression and the estimated infiltration value of CAFs for the tumor tissues of BRCA-LumA, ESCA, HNSC-HPV-, KIRC, KIRP, MESO, PAAD, PCPG and THCA was observed statistically positive (Fig. 5A). However, it showed negative correlation in the tumor tissues of HNSC-HPV+ (Fig. 5A). The scatterplot data were shown by selecting the algorithm with the most statistically significant results. For instance, the SPDL1 expression in ESCA was positively correlated with the infiltration level of CAFs (Fig. 5B, cor = 0.254, P = 5.88e−04) based on the EPIC algorithm.

### Functional enrichment analysis

**Fig. 6:**
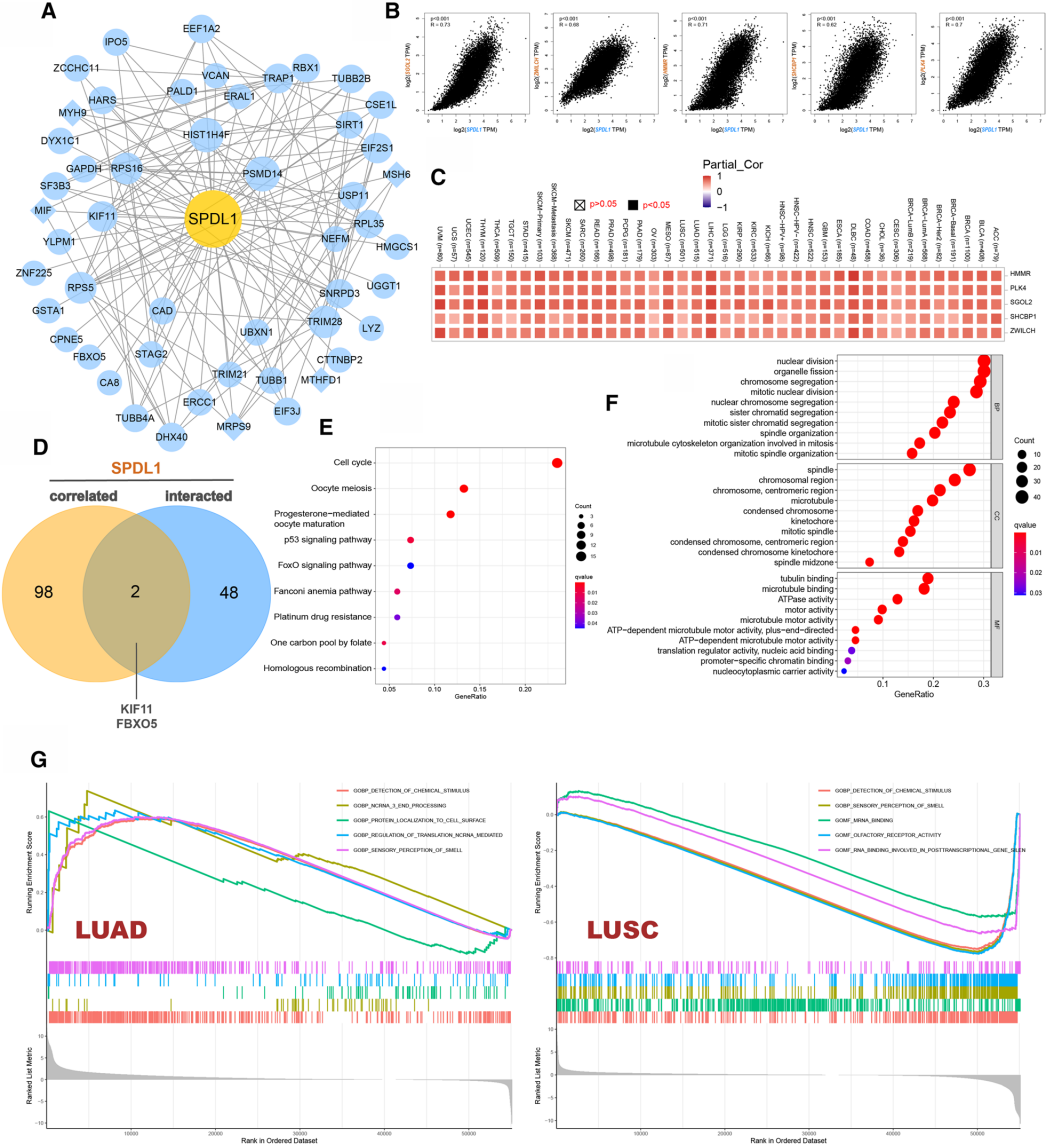
Enrichment analysis of SPDL1. **A** PPI network of top 50 genes related to the expression of SPDL1. **B** The top 100 SPDL1-correlated genes in TCGA and the correlation between SPDL1 and selected targeting genes, including SGOL2, ZWILCH, HMMR, SHCBP1, PLK4. **C** The corresponding heatmap in the cancer types of TCGA. **D** The analysis of the SPDL1 interaction and correlated genes. **E** Bubble plot for KEGG enrichment analysis. **F** Bubble plot for GO enrichment analysis of top 10 terms. **G** GSEA for SPDL1 in LUAD and LUSC based on GO gene sets. P < .05 was considered significant. KEGG: Kyoto encyclopedia of genes and genomes; GO: Gene Ontology; BP: biological processes; CC: cell component; MF: molecular function. GSEA: Gene set enrichment analysis

Analyzing the functional interactions between proteins may provide insights into the mechanisms of formation and progression of cancers. We obtained 50 SPDL1-related proteins by STRING tool. PPI network was visualized by the Cytoscape3.8.2 software (Fig. 6A). The top 100 genes related with SPDL1 expression of all tumor tissues in TCGA were obtained by the GEPIA2 tool. As shown in Fig. 6B, the positive correlations between the expression of SPDL1 and the top five corelated genes were presented by scatter plots, including SGOL2 (Shugoshin 2) (R = .73), ZWILCH (zwilch kinetochore protein) (R = .68), HMMR (hyaluronan mediated motility receptor) (R = .71), SHCBP1(SHC binding and spindle associated 1) (R = .62) and PLK4 (polo like kinase 4) (R = .7). The SPDL1 expression level was positively correlated with the above five genes in all cancer types of TCGA (Fig. 6C).

Based on Venn Diagrams, we obtained two shared genes, KIF11 and FBXO5, by comparing the SPDL1 correlated and interacted genes (Fig. 6D). The above 50 SPDL1 related proteins and top 100 related genes was used for pathway enrichment analysis. After combining the two datasets, we performed KEGG and GO enrichment analysis. The top five enriched pathways obtained in the KEGG pathway analysis were “Cell cycle”, “Oocyte meiosis”, “Progesterone-mediated oocyte maturation”, “p53 signaling pathway” and “FoxO signaling pathway”. The GO enrichment analysis showed that the genes were mainly involved in nuclear division, organelle fission and chromosome segregation (Fig. 6E, F).

### Gene set enrichment analysis

SPDL1-related genes were analyzed through GSEA to identify pathways that were activated in all cancer types of TCGA between high and low SPDL1 expression. In LUAD, detection of chemical stimulus, ncRNA3 end processing, protein localization to cell surface, regulation of translation ncRNA mediated and sensory perception of smell were enriched in the high SPDL1 expression groups. In LUSC, GO terms enriched in the high SPDL1 phenotype mainly contained detection of chemical stimulus, sensory perception of smell, mRNA binding, olfactory receptor activity and RNA binding involved in posttranscriptional gene silencing (Fig. 6G).

## Discussion

SPDL1 is considered to play an important role in the cell cycle, which is associated with the progression of many cancers. As far as we know, there is no information available about the analysis of SPDL1 in pan-cancers. Thus, in this study we have analyzed the molecular features of SPDL1, such as gene expression, genetic alteration, protein expression, methylation level, protein phosphorylation, immune infiltration, survival prognosis, and gene enrichment analysis in different cancer types.

Spindly is reported to be overexpressed in lung cancer cells [19], and we confirmed this through a public online database. For lung cancer in TCGA, the relationship between survival prognosis and SPDL1 expression was detected, and we found that high SPDL1 expression was related with poor OS and DFS in LUAD (P = .0015, P = .03, respectively) but not in LUSC (P>.05) (Fig. 2) based on the GEPIA2 tool. Additionally, we conducted a survival analysis in 865 LUAD and 675 LUSC, and found that high SPDL1 expression was associated with poor OS, PPS in LUAD, and poor FP in LUSC using the Kaplan-Meier plotter tool (Additional file 3: Table S2). We then performed IHC in LUAD and found a statistical correlation between high SPDL1 expression and poor OS (P = .03841). In conclusion, our results revealed that the high SPDL1 expression in lung cancer was linked to poor OS. Tumor-infiltrating CD8+ T cells have a vital effect on the immune response in lung cancer [38–40]. Hence, several immune-related prognostic models have been developed [41, 42]. SPDL1 was regarded as one of the genes associated with CD8+ T cells infiltration in the early stage of LUAD, which may provide valuable predictions for the survival risk of patients [43]. However, we did not find the significant correlation between SPDL1 expression and the immune infiltration level of CD8+ T cells in all stages of LUAD with different algorithms, but we found it to be statistically significant in LUSC (Additional file 2: Fig. S10). In LUAD, the expression of SPDL1 is significantly related to TMB, HRD and aneuploidy and may be involved in the tumorigenesis of LUAD.

As shown in the KEGG pathway analysis, SPDL1 was associate with “Oocyte meiosis”. Knockdown of Spindly restored the asymmetric division of oocytes, which was a normal process in mammalian before fertilization [44]. We next explored the role of SPDL1 in the reproductive system. Notably, we found that only the comparison of SPDL1 phosphorylation levels in S555 with OV showed the significant differences (Additional file 2: Fig. S9A). In contrast, there was no association between SPDL1 expression and genomic instability in OV. The effect of SPDL1 phosphorylation on OV tumorigenesis needs further investigation. We observed that high expression of SPDL1 was associated with poor OS, RFS, PPS in ovarian cancer, especially in subgroups, such as “grade 4”, “stage I, III, IV” (Additional file 3: Table S3). More experiments are needed to confirm the roles of SPDL1 in prognosis of ovarian cancer. Survival analysis showed a correlation between high SPDL1 expression and poor OS in UCEC. Compared to UCEC cases without SPDL1 alteration, DSS and PFS were better in cases with SPDL1 alteration, but DFS and OS were worse. Enhanced RNA tissue specificity was shown in the normal testis samples. Moreover, SPDL1 expression was not significantly correlated with genomic instability and the survival rate in TGCT. All in all, the SPDL1 gene may not be associated with the development of the TGCT.

A previous study reported that SPDL1 was a tumor suppressor gene for colorectal cancer (CRC), which acted in the downstream of MRTFB to regulate CRC growth [18]. In our work, there was no significant correlation between survival and SPDL1 expression in COAD and READ, but it showed a trend that lower SPDL1 expression levels were associated with poor prognosis. The expression of SPDL1 was associated with TMB, MSI, HRD in COAD and READ. The hub gene set variation analysis (HGSVA) score of the gene set, which include the SPDL1, may reflect the pathological progression from liver cirrhosis to HCC, and SPDL1 expression was an independent prognostic factor for both OS and RFS [45]. In our research, it showed a correlation between high SPDL1 expression and poor OS and DFS in LIHC. Based on the Kaplan-Meier plotter, a high SPDL1 expression level was associated with poor OS, DMFS, PPS, RFS in breast cancer. Interestingly, when we analyzed the patients with negative ER and positive PR of breast cancer, SPDL1 low expression is significantly correlated with poor OS. Hence, we should also focus on the other clinical characteristics. Notably, in contrast with most of other tumors, a low expression level was linked to poor OS, FP, PPS in gastric cancer (P < .05). This suggested that the SPDL1 gene worked differently in gastric cancer.

To analyze the role of SPDL1 in cancers, we performed an enrichment analysis of SPDL1-related genes and proteins. It was involved in “Cell cycle” and “Platinum drug resistance” based on KEGG analysis, and there was a publication used SPDL1 to detect cell cycle progression [46]. This finding was also consistent with the previous analysis that inhibiting of Spindly was cytotoxic to oral squamous cell carcinoma (OSCC) cells and increased its chemical sensitivity to cisplatin [15]. Paclitaxel, one of the Microtubule-targeting agents (MTAs), delays cells in mitosis which could lead to cell death in mitosis (DiM) through the accumulation of an apoptotic signal [47, 48]. Insufficient expression of SPDL1 was observed during paclitaxel resistance, and spindle inhibition increased the efficacy of low-dose paclitaxel [14, 19]. Above all, we need to study the potential therapeutic benefits of combining SPDL1 inhibition with platinum drug.

Although the correlations of SPDL1 expression with survival prognosis, genetic alteration, genomic instability, DNA methylation, protein phosphorylation and immune infiltration were computationally explored and analyzed, some limitations should be acknowledged. Firstly, the experiment was only conducted in LUAD tissues, but not in other tumors. The second limitation is the lack of both the molecular and cellular mechanism of the SPDL1 expression. In the future, more experiments and large-scale clinical trials are needed to further validate these findings and to explore the follow-ups of functional mechanisms of SPDL1 in cancers.

## Conclusions

In this pan-cancer study, we provided a comprehensive and systematic characterization of SPDL1, and demonstrated the oncogenic role of SPDL1 in cancers. In summary, SPDL1 was widely overexpressed in most cancer types, meanwhile its overexpression is mostly associated with poor prognosis, which indicated that SPDL1 may promote tumorigenesis and play a pivotal role as a potential oncogene. Moreover, our results indicated that there is a potential therapeutic benefit of combining SPDL1 inhibition with platinum drug.

## Supplementary Information

**Additional file 1.** Supplementary materials and methods. 1.1 Gene mapping and protein structure analysis. 1.2 Gene expression analysis in HPA. 1.3 Gene expression analysis of Oncomine. 1.4 Phosphorylation feature prediction. 1.5 DNA methylation analysis. 1.6 Survival prognosis analysis of Kaplan-Meier plotter. 

**Additional file 2: Fig. S1.** Structural characteristics of SPDL1. (A). Genomic location of human SPDL1. (B). SPDL1 protein structure with conserved domains among ten species. **Fig. S2.** The evolutionary relationship of SPDL1 protein among different species were shown with the phylogenetic tree. **Fig. S3.** SPDL1 expression in different cells and tissues of normal physiological samples. (A). The SPDL1 gene expression in different tissues were analyzed by using the consensus datasets of HPA, GTEx and FANTOM5. (B). The SPDL1 gene expression in different blood cells were analyzed by using the consensus dataset of HPA, Monaco and Schmiedel. **Fig. S4.** SPDL1 expression in different cancer types. (A) There was no significant difference in the SPDL1 expression between tumor tissues and the normal tissues in ACC, OV, TGCT, UCS. (B) SPDL1 expression in different pathological stages. **Fig. S5.** Using the Oncomine database, we analyzed the SPDL1 expression between normal and tumor tissues (A). Cervical Cancer. (B). Head and Neck Cancer. (C). Colorectal Cancer. (D) Lung Cancer. (E) Sarcoma. (F). Kidney Cancer. **Fig. S6.** Correlation between SPDL1 gene expression and survival prognosis, including OS, DSS, PFS, RFS, DMFS, PPS and FP, of cancers using the Kaplan-Meier plotter. (A). Liver Cancer. (B). Breast Cancer. (C). Gastric Cancer. (D). Lung Cancer. (E). Ovarian Cancer. **Fig. S7.** The forest figure showed the correlation between SPDL1 expression and prognosis of liver cancer, breast cancer, gastric cancer, lung cancer and ovarian cancer. **Fig. S8.** The level of SPDL1 methylation in different tumors and its association with gene expression. (A,B) Promoter methylation levels of SPDL1 in different cancer types compared with normal tissues. (C). The level of SPDL1 methylation with probes were analyzed using the MEXPRESS. The beta value of methylation, the Benjamini-Hochberg-adjusted P-value and the Pearson correlation coefficients (R) were displayed. **Fig. S9.** Phosphorylation analysis of SPDL1 protein via the UALCAN. (A). The phosphoprotein site with significant differences. (B). The Expression level of SPDL1 phosphoprotein (NP_060255.3 site) between normal tissue and primary tissue of breast cancer and ovarian cancer in the box plots. (C). The detail information of S555 based on the PhosphoNET database. **Fig. S10.** Correlation between the SPDL1 gene expression and immune infiltration of CD8+ T-cells in all cancer types of TCGA. (A). Different algorithms (EPIC, MCPCOUNTER, CIBERSORT-ABS and QUANTISEQ) were used to explore the correlation between the infiltration level of CD8+ T-cells and the SPDL1 gene expression in all cancer types of TCGA. (B). The scatterplot data of the selected algorithm with the most statistically significant results. 

**Additional file 3:** **Table S1.** Subgroup analysis on the correlation of SPDL1 expression and prognosis of breast cancer cases. **Table S2.** Subgroup analysis on the correlation of SPDL1 expression and prognosis of lung cancer cases. **Table S3.** Subgroup analysis on the correlation of SPDL1 expression and prognosis of ovarian cancer cases. **Table S4.** Subgroup analysis on the correlation of SPDL1 expression and prognosis of gastric cancer cases. **Table S5.** Subgroup analysis on the correlation of SPDL1 expression and prognosis of liver cancer cases.

## Data Availability

The data sets used and/or analyzed during the current study are available from the corresponding author on reasonable request.
