# Supplementary material for: Pan-cancer analysis combined with experiments explores the oncogenic role of spindle apparatus coiled-coil protein 1 (SPDL1)
Source: Cancer Cell Int. 2022 Jan 29;22:49. doi: 10.1186/s12935-022-02461-w (PMC8801078; doi:10.1186/s12935-022-02461-w)
Supplement: Supplementary file 1 — Additional file 1. Supplementary materials and methods. 1.1 Gene mapping and protein structure analysis. 1.2 Gene expression analysis in HPA. 1.3 Gene expression analysis of Oncomine. 1.4 Phosphorylation feature prediction. 1.5 DNA methylation analysis. 1.6 Survival prognosis analysis of Kaplan-Meier plotter. [file 12935_2022_2461_MOESM1_ESM.docx]

# Supplementary data

# 1. Supplementary materials and methods

## 1.1 Gene mapping and protein structure analysis

We used Ensembl, which is a genome browser for vertebrate genomes that supports research in comparative genomics, evolution, sequence variation and transcriptional regulation (<http://asia.ensembl.org/index.html>) to obtain the genome location information of the SPDL1 gene of human^1^. "HomoloGene" function (<https://www.ncbi.nlm.nih.gov/homologene/>) of the National Center for Biotechnology Information (NCBI) were applied to get the proteins used in sequence comparisons and their conserved domain architectures by rpsblast searching. Subsequently, we downloaded the protein sequences in FASTA format and got the phylogenetic tree with Molecular Evolutionary Genetics Analysis version 11 (MEGA11) ( <https://megasoftware.net/>)^2^.

## 1.2 Gene expression analysis in HPA

The online Human protein atlas (HPA) database (<https://www.proteinatlas.org/humanproteome/pathology>) were used to obtain the expression data of the SPDL1 gene in cells, tissues and organs using an integration of omics technologies. The Tissue Atlas shows Consensus Normalized eXpression (NX) levels for 55 tissue types and 6 blood cell types, by combining the data from the three datasets, including HPA, GTEx and FANTOM5. The Blood Atlas shows the resulting transcript expression values, denoted Normalized eXpression (NX), resulting from the internal normalization pipeline for 18 blood cell types, including various B- and T-cells, monocytes, granulocytes and dendritic cells. The consensus blood cell type data contains the data from HPA, data from 15 blood cell types by Schmiedel et al.^3^ and 29 blood cell types as well as total PBMC by Monaco et al^4^. Enhanced is defined as NX levels of a group (of 1-5 tissues or 1-10 cell types or 1-5 brain regions) at least four times the mean of other tissue/region/cell types. “Low specificity” means NX ≥ 1 in at least one tissue/region/cell type but not elevated in any tissue/region/cell type.

## 1.3 Gene expression analysis of Oncomine

We used the Oncomine database^5^ (<https://www.oncomine.org/resource/login.html> ) and analyzed the difference of SPDL1 expression between cancer and normal tissues by choosing “Cancer vs. Normal ”analysis type, entering “SPDL1” and setting the P-value =0.05, fold change =1.5. Then, we compared data for different cancer type. It showed the rank for a gene which is the median rank for that gene across each of the analyses. The p-Value for a gene is its p-Value for the median-ranked analysis. It presents the legends of the studies that were screened out.

## 1.4 Phosphorylation feature prediction

The UALCAN data portal (<http://ualcan.path.uab.edu/analysis-prot.html>) now provides protein expression analysis option using data from Clinical Proteomic Tumor Analysis Consortium (CPTAC) Confirmatory/Discovery dataset^6^. Here, utilizing the Clinical Proteomic Tumor Analysis Consortium (CPTAC) mass-spectrometry-based proteomics datasets, we explored the expression level of the total protein and phosphoprotein (with phosphorylation at the S555 site) of SPDL1 (NP_001316568.1) between primary tumor and normal tissues, respectively, by entering “SPDL1”. Two kinds of tumors were available, namely, breast cancer and ovarian cancer. We logged into the PhosphoNET database (<http://www.phosphonet.ca/>), and obtained the predicted phosphorylation features of the S555 site by searching “SPDL1”.

## 1.5 DNA methylation analysis

Using the “TCGA gene analysis” module of the UALCAN portal^7^ (<http://ualcan.path.uab.edu/analysis-prot.html>), we explored the difference in promotor methylation levels of SPDL1 between primary tumor and normal tissues in all tumor types of TCGA. The Beta value indicates level of DNA methylation ranging from 0 (unmethylated) to 1 (fully methylated). Different beta value cut-off has been considered to indicate hyper-methylation [Beta value: 0.7 - 0.5] or hypo-methylation [Beta-value: 0.3-0.25]. We logged into the MEXPRESS website (https://mexpress.be/)^8,9^ and query “SPDL1” to investigate the DNA methylation level of SPDL1 in LUAD and LUSC. The Benjamini-Hochberg-adjusted P-value and Pearson’s correlation coefficient (R) value were obtained.

## 1.6 Survival prognosis analysis of Kaplan-Meier plotter

The Kaplan-Meier plotter (<http://kmplot.com/analysis/>) were used to perform survival prognosis analysis, which contained OS, distant metastasis-free survival (DMFS), relapse-free survival (RFS), post-progression survival (PPS), first progression (FP), disease-specific survival (DSS), and progress-free survival (PFS), by the different GEO datasets ,including breast, lung, ovarian, gastric, and liver cancers. The patients were split into two groups by selecting “autoselect best cutoff”. It computed the hazard ratio (HR), 95% confidence intervals and log-rank P-value. The Kaplan-Meier survival plots were generated. We put the results above altogether and make a forest map. In addition, we filtered the clinical factors for a series of subgroup analyses.

# 2. Supplementary figure legends

Fig. S1. Structural characteristics of SPDL1. (A). Genomic location of human SPDL1. (B). SPDL1 protein structure with conserved domains among ten species.

Fig. S2. The evolutionary relationship of SPDL1 protein among different species were shown with the phylogenetic tree.

Fig. S3. SPDL1 expression in different cells and tissues of normal physiological samples. (A). The SPDL1 gene expression in different tissues were analyzed by using the consensus datasets of HPA, GTEx and FANTOM5. (B). The SPDL1 gene expression in different blood cells were analyzed by using the consensus dataset of HPA, Monaco and Schmiedel.

Fig. S4. SPDL1 expression in different cancer types. (A) There was no significant difference in the SPDL1 expression between tumor tissues and the normal tissues in ACC, OV, TGCT, UCS. (B) SPDL1 expression in different pathological stages.

Fig. S5. Using the Oncomine database, we analyzed the SPDL1 expression between normal and tumor tissues (A). Cervical Cancer. (B). Head and Neck Cancer. (C). Colorectal Cancer. (D) Lung Cancer. (E) Sarcoma. (F). Kidney Cancer.

Fig. S6. Correlation between SPDL1 gene expression and survival prognosis, including OS, DSS, PFS, RFS, DMFS, PPS and FP, of cancers using the Kaplan-Meier plotter. (A). Liver Cancer. (B). Breast Cancer. (C). Gastric Cancer. (D). Lung Cancer. (E). Ovarian Cancer.

Fig. S7. The forest figure showed the correlation between SPDL1 expression and prognosis of liver cancer, breast cancer, gastric cancer, lung cancer and ovarian cancer.

Fig. S8. The level of SPDL1 methylation in different tumors and its association with gene expression. (A,B) Promoter methylation levels of SPDL1 in different cancer types compared with normal tissues. (C). The level of SPDL1 methylation with probes were analyzed using the MEXPRESS. The beta value of methylation, the Benjamini-Hochberg-adjusted P-value and the Pearson correlation coefficients (R) were displayed.

Fig. S9. Phosphorylation analysis of SPDL1 protein via the UALCAN. (A). The phosphoprotein site with significant differences. (B). The Expression level of SPDL1 phosphoprotein (NP_060255.3 site) between normal tissue and primary tissue of breast cancer and ovarian cancer in the box plots. (C). The detail information of S555 based on the PhosphoNET database.

Fig. S10. Correlation between the SPDL1 gene expression and immune infiltration of CD8+ T-cells in all cancer types of TCGA. (A). Different algorithms (EPIC, MCPCOUNTER, CIBERSORT-ABS and QUANTISEQ) were used to explore the correlation between the infiltration level of CD8+ T-cells and the SPDL1 gene expression in all cancer types of TCGA. (B). The scatterplot data of the selected algorithm with the most statistically significant results.

# 3. Supplementary table

Table S1. Subgroup analysis on the correlation of SPDL1 expression and prognosis of breast cancer cases.

Table S2. Subgroup analysis on the correlation of SPDL1 expression and prognosis of lung cancer cases.

Table S3. Subgroup analysis on the correlation of SPDL1 expression and prognosis of ovarian cancer cases.

Table S4. Subgroup analysis on the correlation of SPDL1 expression and prognosis of gastric cancer cases.

Table S5. Subgroup analysis on the correlation of SPDL1 expression and prognosis of liver cancer cases.

1. Howe KL, Achuthan P, Allen J, et al: Ensembl 2021. Nucleic Acids Res 49:D884-D891, 2021

2. Tamura K, Stecher G, Kumar S: MEGA11: Molecular Evolutionary Genetics Analysis Version 11. Mol Biol Evol 38:3022-3027, 2021

3. Schmiedel BJ, Singh D, Madrigal A, et al: Impact of Genetic Polymorphisms on Human Immune Cell Gene Expression. Cell 175:1701-1715 e16, 2018

4. Monaco G, Lee B, Xu W, et al: RNA-Seq Signatures Normalized by mRNA Abundance Allow Absolute Deconvolution of Human Immune Cell Types. Cell Rep 26:1627-1640 e7, 2019

5. Daniel R Rhodes JY, K Shanker, Nandan Deshpande, Radhika Varambally, Debashis Ghosh, Terrence Barrette, Akhilesh Pandey, Arul M Chinnaiyan: ONCOMINE: a cancer microarray database and integrated data-mining platform. Neoplasia 6, 2004

6. Monsivais D, Vasquez YM, Chen F, et al: Mass-spectrometry-based proteomic correlates of grade and stage reveal pathways and kinases associated with aggressive human cancers. Oncogene 40:2081-2095, 2021

7. Chandrashekar DS, Bashel B, Balasubramanya SAH, et al: UALCAN: A Portal for Facilitating Tumor Subgroup Gene Expression and Survival Analyses. Neoplasia 19:649-658, 2017

8. Koch A, De Meyer T, Jeschke J, et al: MEXPRESS: visualizing expression, DNA methylation and clinical TCGA data. BMC Genomics 16:636, 2015

9. Koch A, Jeschke J, Van Criekinge W, et al: MEXPRESS update 2019. Nucleic Acids Res 47:W561-w565, 2019
