# Supplementary material for: Pan-cancer analysis combined with experiments explores the oncogenic role of spindle apparatus coiled-coil protein 1 (SPDL1)
Source: Cancer Cell Int. 2022 Jan 29;22:49. doi: 10.1186/s12935-022-02461-w (PMC8801078; doi:10.1186/s12935-022-02461-w)
Supplement: Supplementary file 2 — Additional file 2: Fig. S1. Structural characteristics of SPDL1. (A). Genomic location of human SPDL1. (B). SPDL1 protein structure with conserved domains among ten species. Fig. S2. The evolutionary relationship of SPDL1 protein among different species were shown with the phylogenetic tree. Fig. S3. SPDL1 expression in different cells and tissues of normal physiological samples. (A). The SPDL1 gene expression in different tissues were analyzed by using the consensus datasets of HPA, GTEx and FANTOM5. (B). The SPDL1 gene expression in different blood cells were analyzed by using the consensus dataset of HPA, Monaco and Schmiedel. Fig. S4. SPDL1 expression in different cancer types. (A) There was no significant difference in the SPDL1 expression between tumor tissues and the normal tissues in ACC, OV, TGCT, UCS. (B) SPDL1 expression in different pathological stages. Fig. S5. Using the Oncomine database, we analyzed the SPDL1 expression between normal and tumor tissues (A). Cervical Cancer. (B). Head and Neck Cancer. (C). Colorectal Cancer. (D) Lung Cancer. (E) Sarcoma. (F). Kidney Cancer. Fig. S6. Correlation between SPDL1 gene expression and survival prognosis, including OS, DSS, PFS, RFS, DMFS, PPS and FP, of cancers using the Kaplan-Meier plotter. (A). Liver Cancer. (B). Breast Cancer. (C). Gastric Cancer. (D). Lung Cancer. (E). Ovarian Cancer. Fig. S7. The forest figure showed the correlation between SPDL1 expression and prognosis of liver cancer, breast cancer, gastric cancer, lung cancer and ovarian cancer. Fig. S8. The level of SPDL1 methylation in different tumors and its association with gene expression. (A,B) Promoter methylation levels of SPDL1 in different cancer types compared with normal tissues. (C). The level of SPDL1 methylation with probes were analyzed using the MEXPRESS. The beta value of methylation, the Benjamini-Hochberg-adjusted P-value and the Pearson correlation coefficients (R) were displayed. Fig. S9. Phosphorylation analysis of SPDL1 prot [file 12935_2022_2461_MOESM2_ESM.docx]

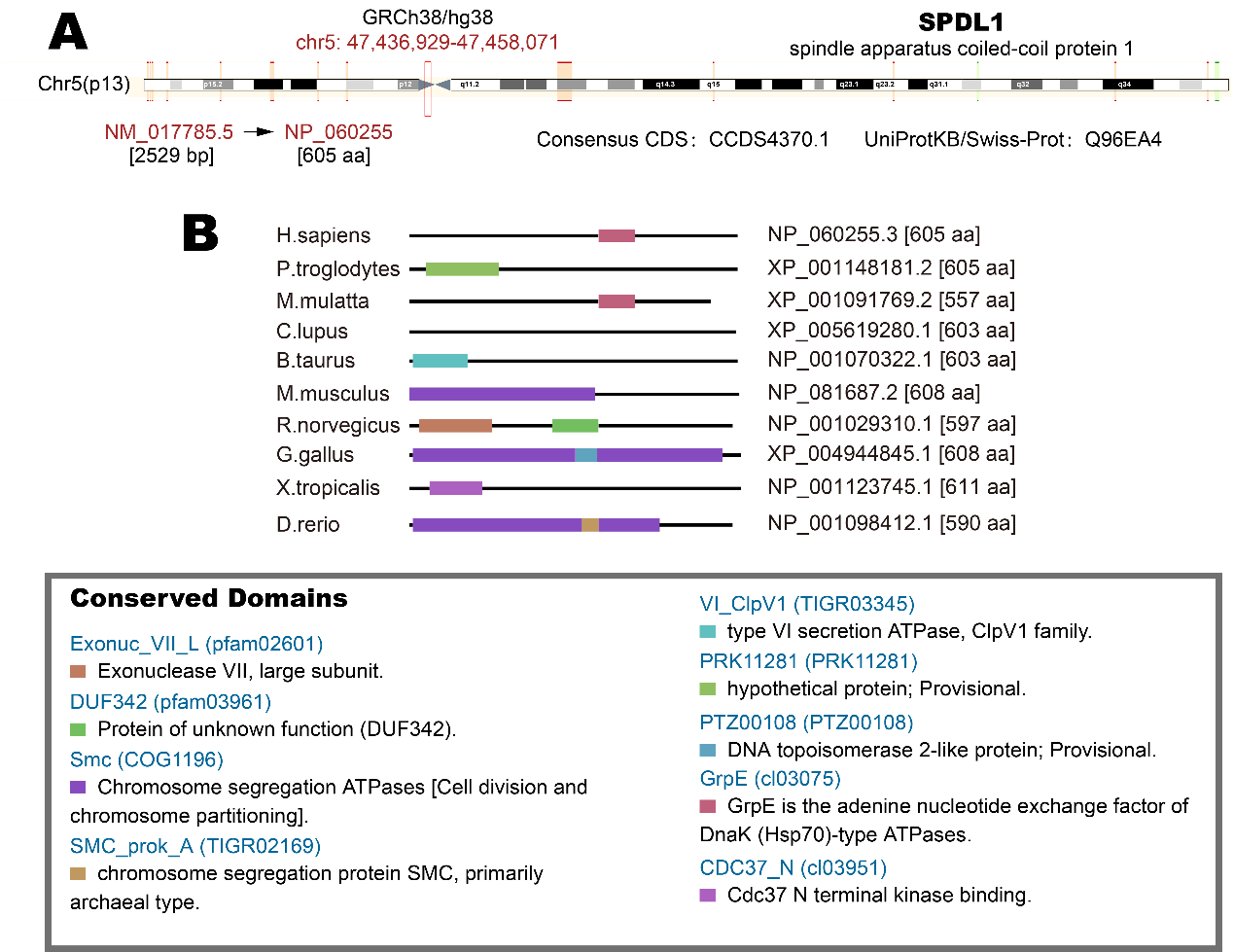
Fig. S1. Structural characteristics of SPDL1. (A). Genomic location of human SPDL1. (B). SPDL1 protein structure with conserved domains among ten species.


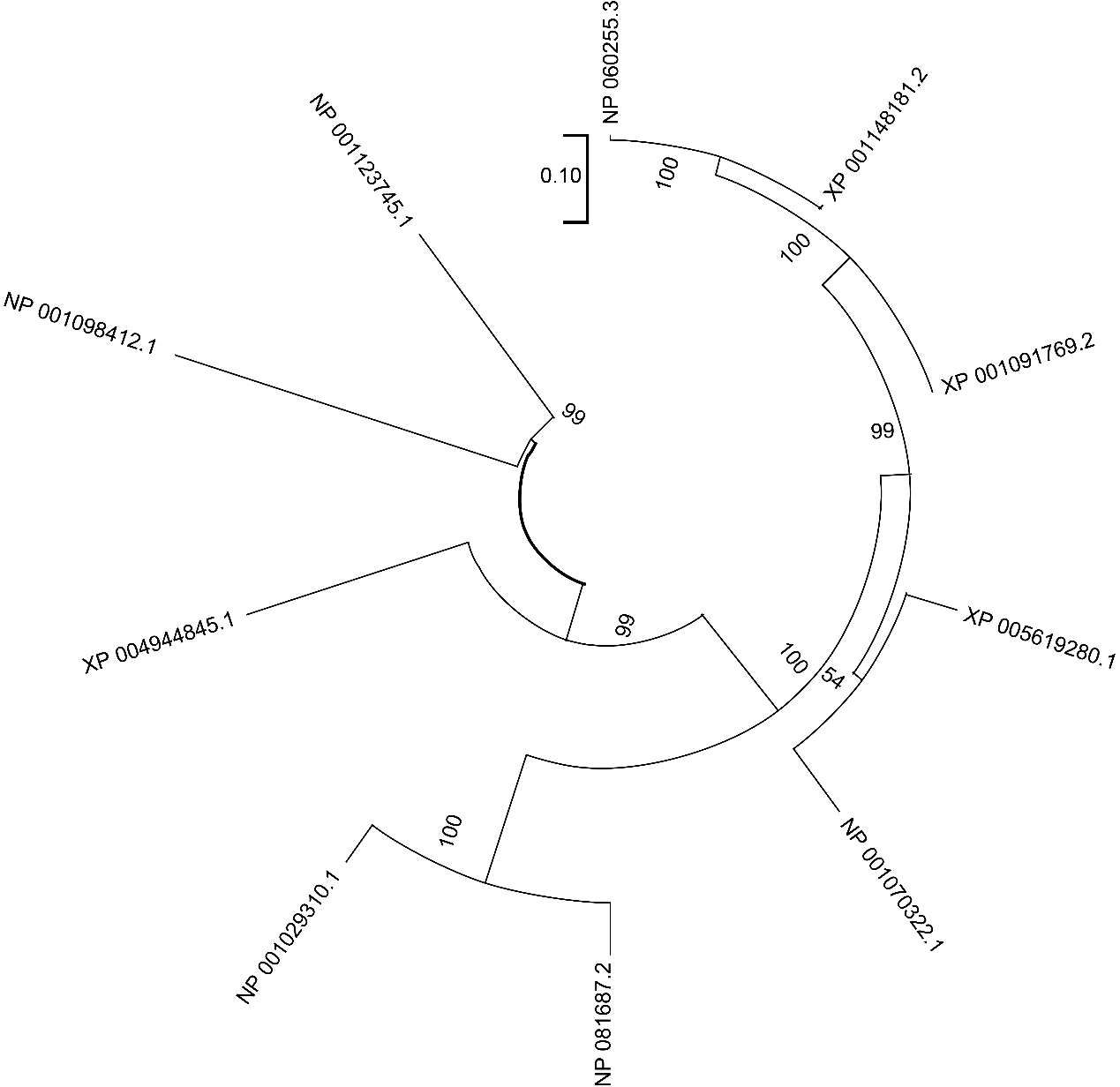


Fig. S2. The evolutionary relationship of SPDL1 protein among different species were shown with the phylogenetic tree.


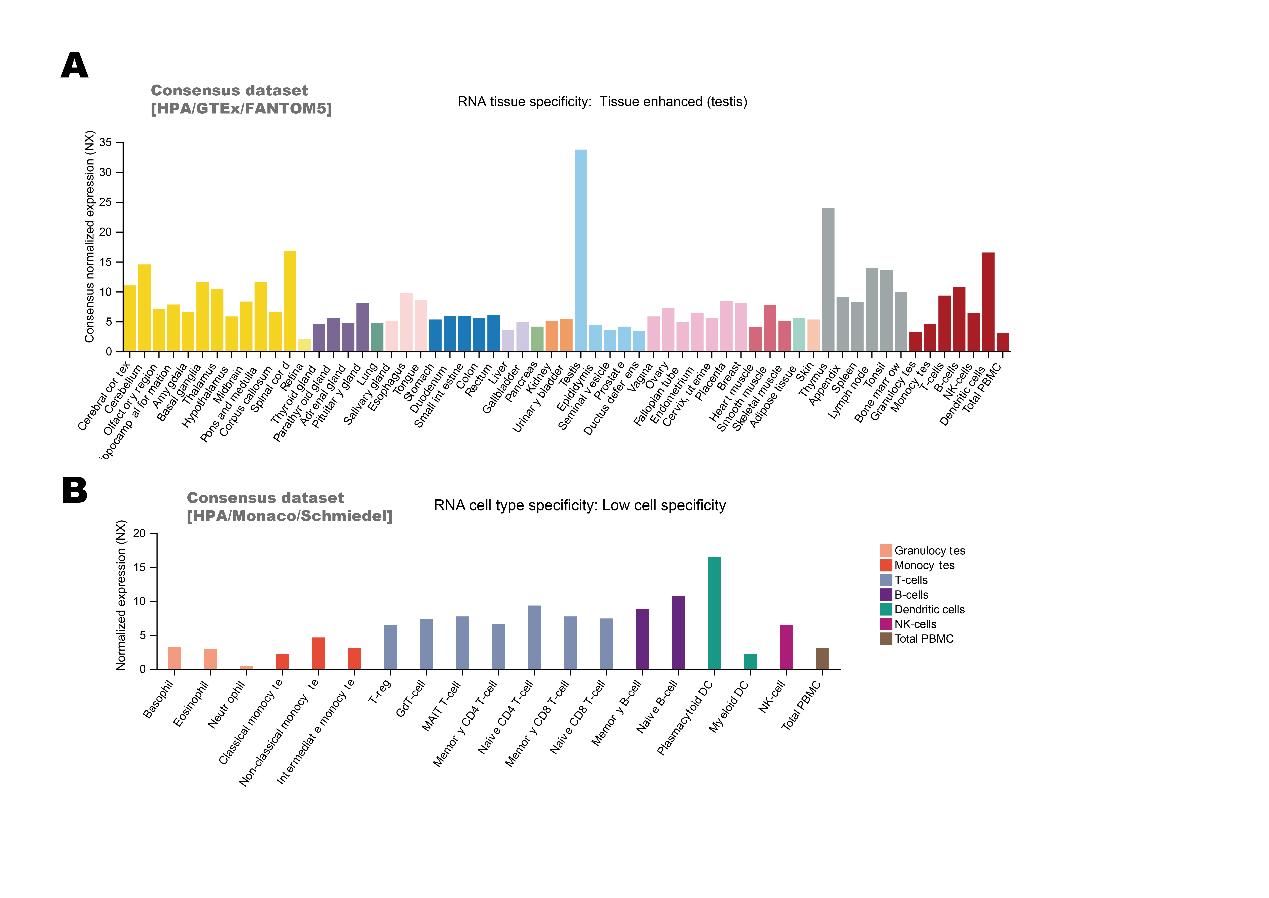


Fig. S3. SPDL1 expression in different cells and tissues of normal physiological samples. (A). The SPDL1 gene expression in different tissues were analyzed by using the consensus datasets of HPA, GTEx and FANTOM5. (B). The SPDL1 gene expression in different blood cells were analyzed by using the consensus dataset of HPA, Monaco and Schmiedel.


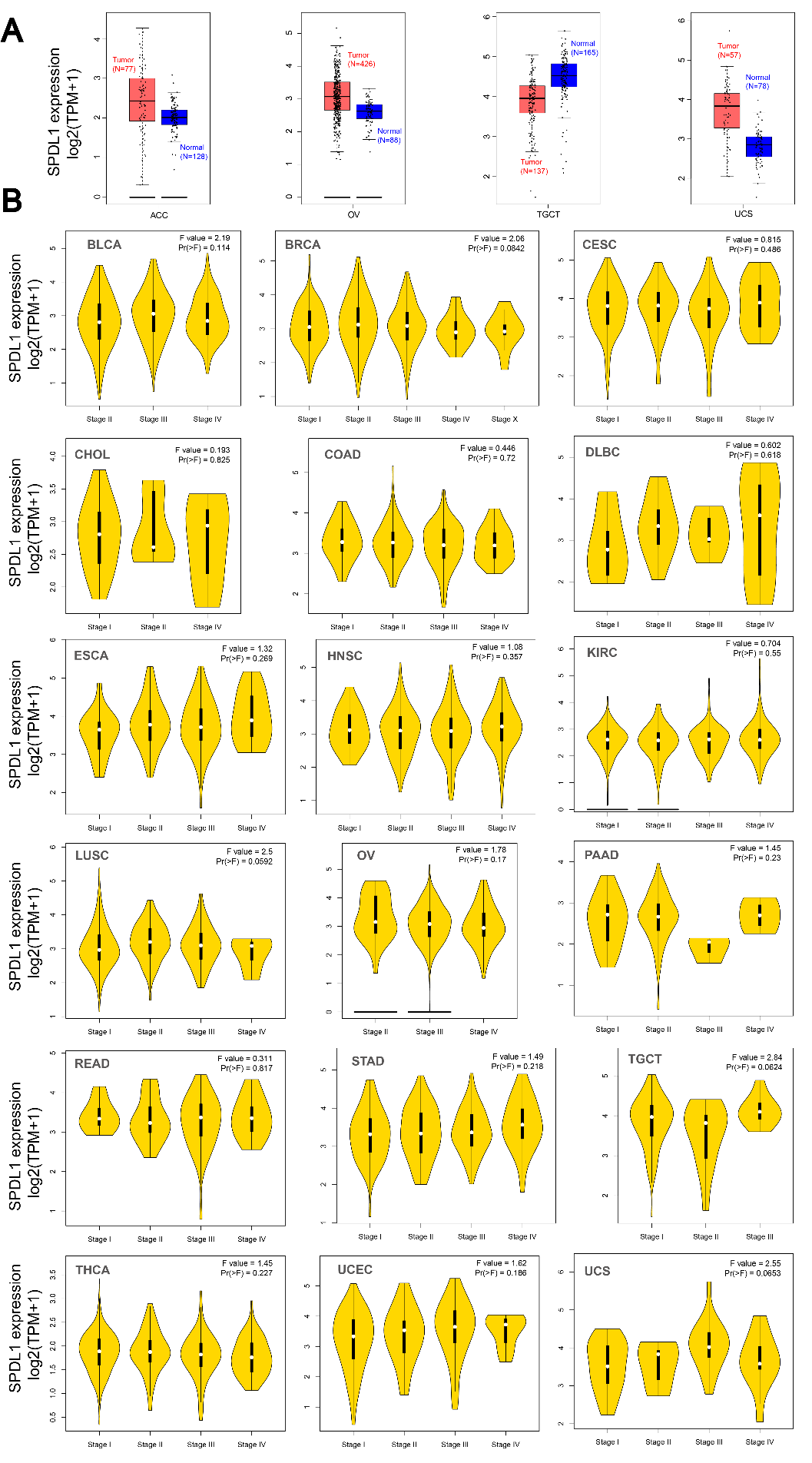


Fig. S4. SPDL1 expression in different cancer types. (A) There was no significant difference in the SPDL1 expression between tumor tissues and the normal tissues in ACC, OV, TGCT, UCS. (B) SPDL1 expression in different pathological stages.


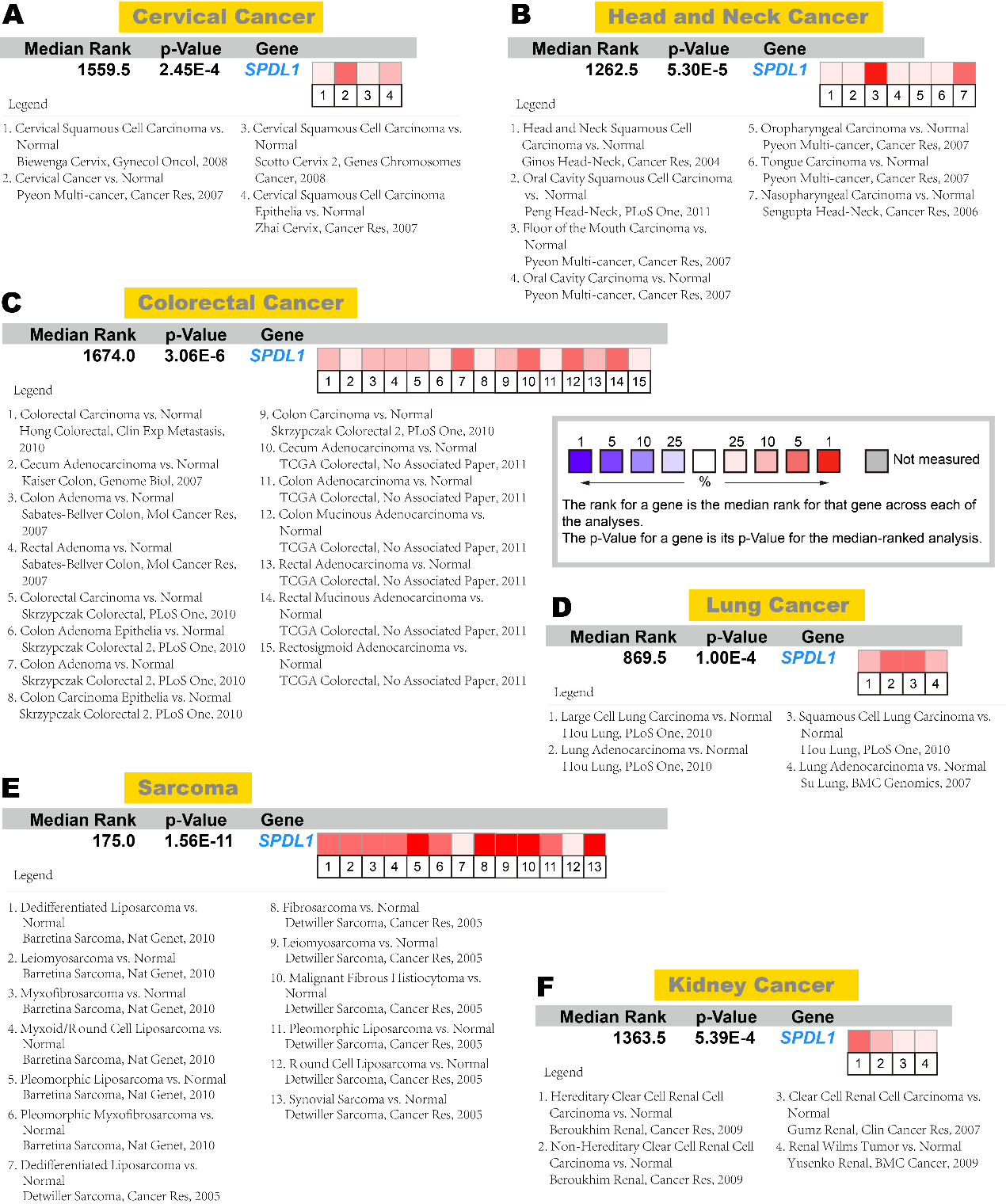


Fig. S5. Using the Oncomine database, we analyzed the SPDL1 expression between normal and tumor tissues (A). Cervical Cancer. (B). Head and Neck Cancer. (C). Colorectal Cancer. (D) Lung Cancer. (E) Sarcoma. (F). Kidney Cancer.


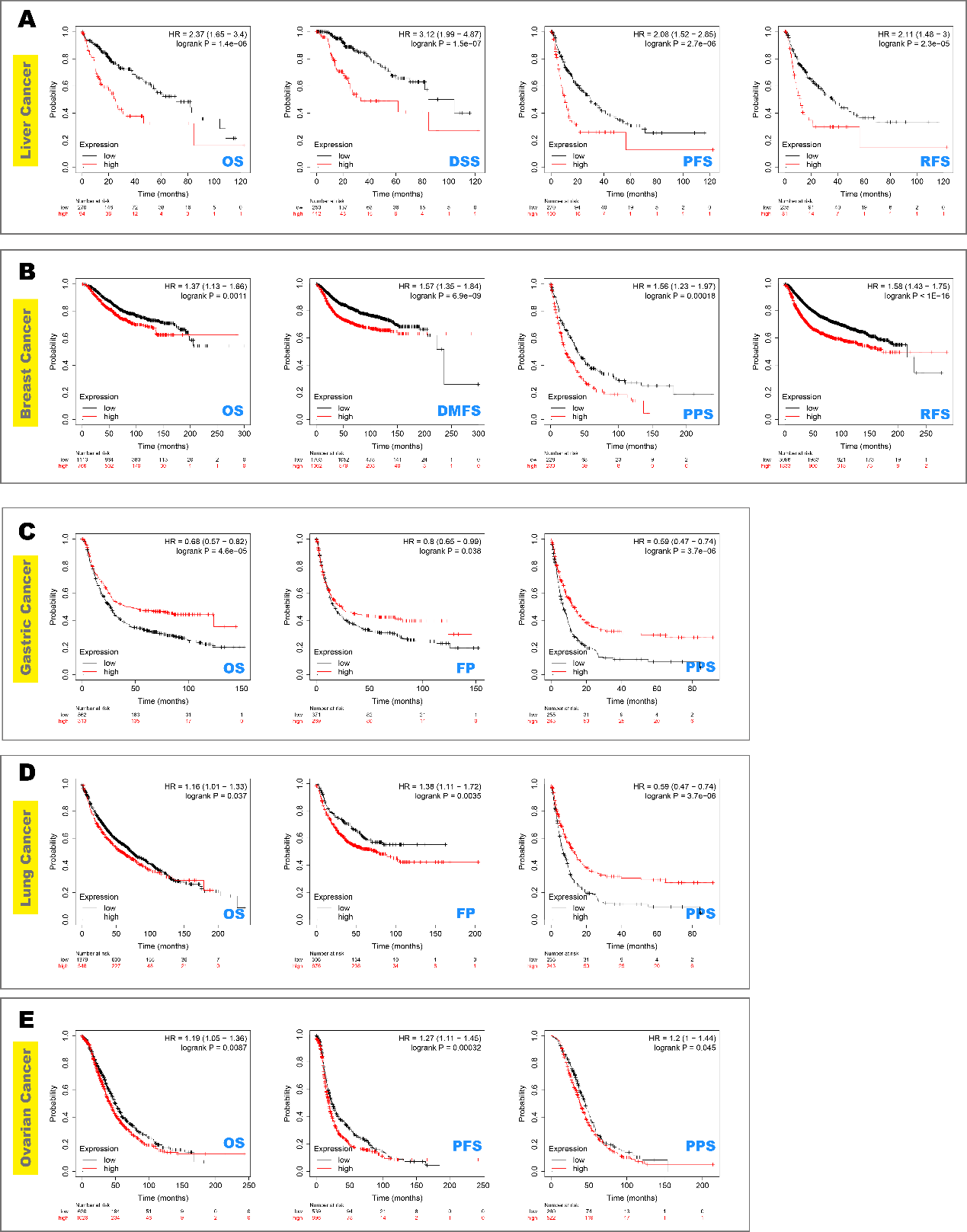


Fig. S6. Correlation between SPDL1 gene expression and survival prognosis, including OS, DSS, PFS, RFS, DMFS, PPS and FP, of cancers using the Kaplan-Meier plotter. (A). Liver Cancer. (B). Breast Cancer. (C). Gastric Cancer. (D). Lung Cancer. (E). Ovarian Cancer.


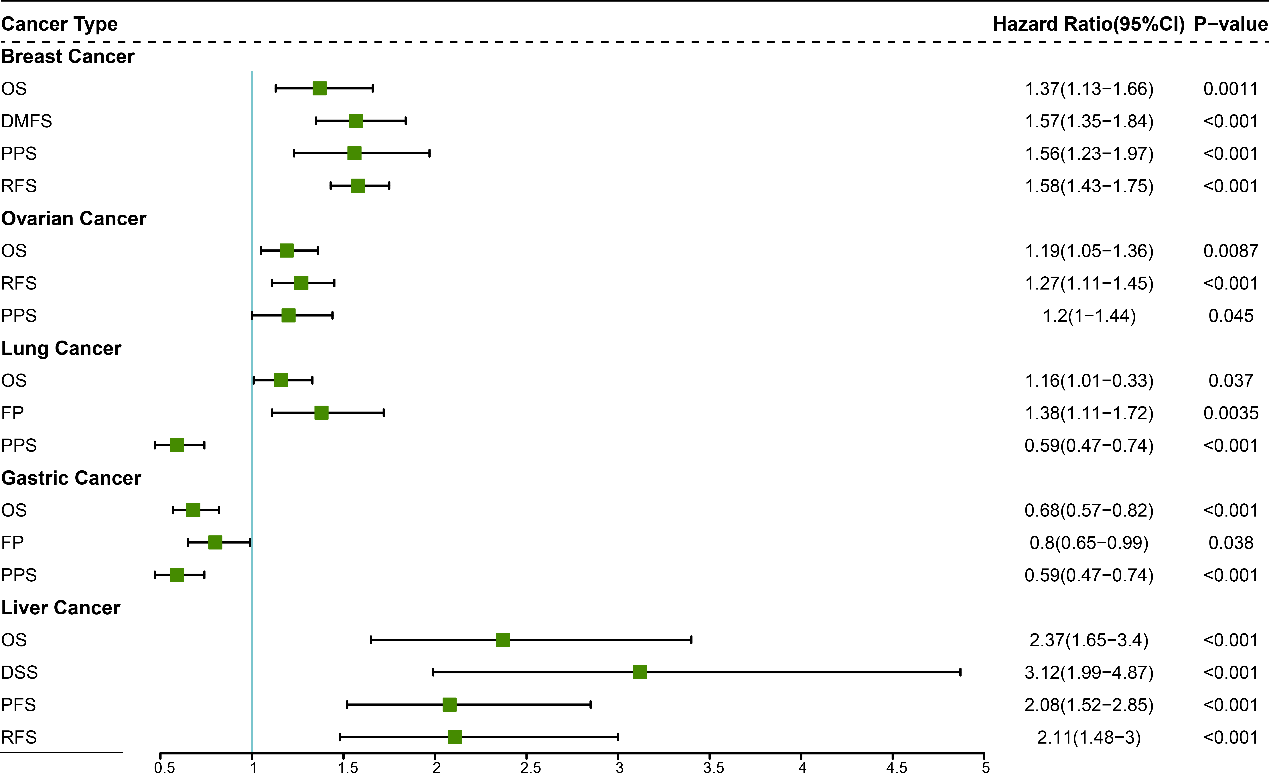


Fig. S7. The forest figure showed the correlation between SPDL1 expression and prognosis of liver cancer, breast cancer, gastric cancer, lung cancer and ovarian cancer.


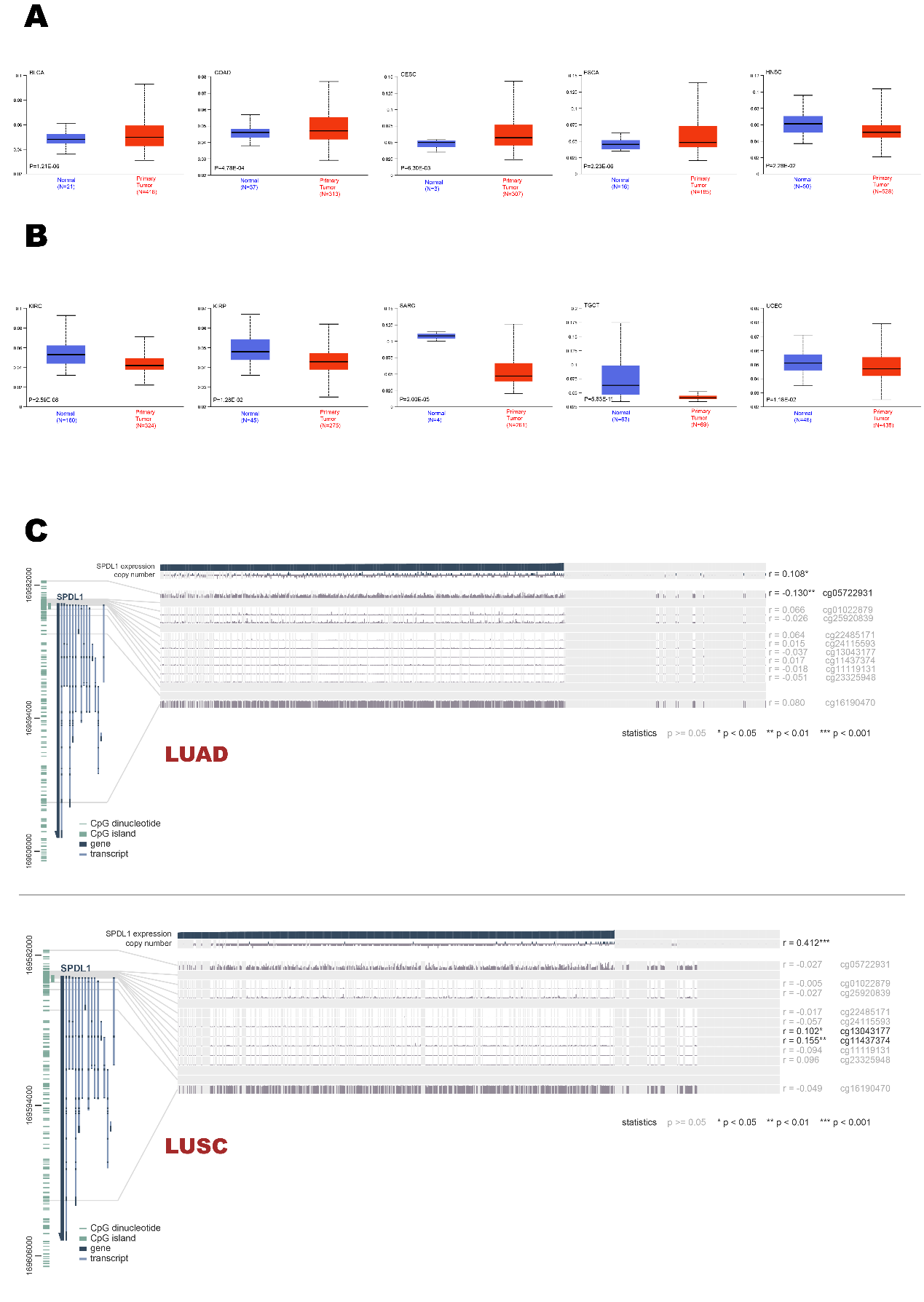


Fig. S8. The level of SPDL1 methylation in different tumors and its association with gene expression. (A,B) Promoter methylation levels of SPDL1 in different cancer types compared with normal tissues. (C). The level of SPDL1 methylation with probes were analyzed using the MEXPRESS. The beta value of methylation, the Benjamini-Hochberg-adjusted P-value and the Pearson correlation coefficients (R) were displayed.


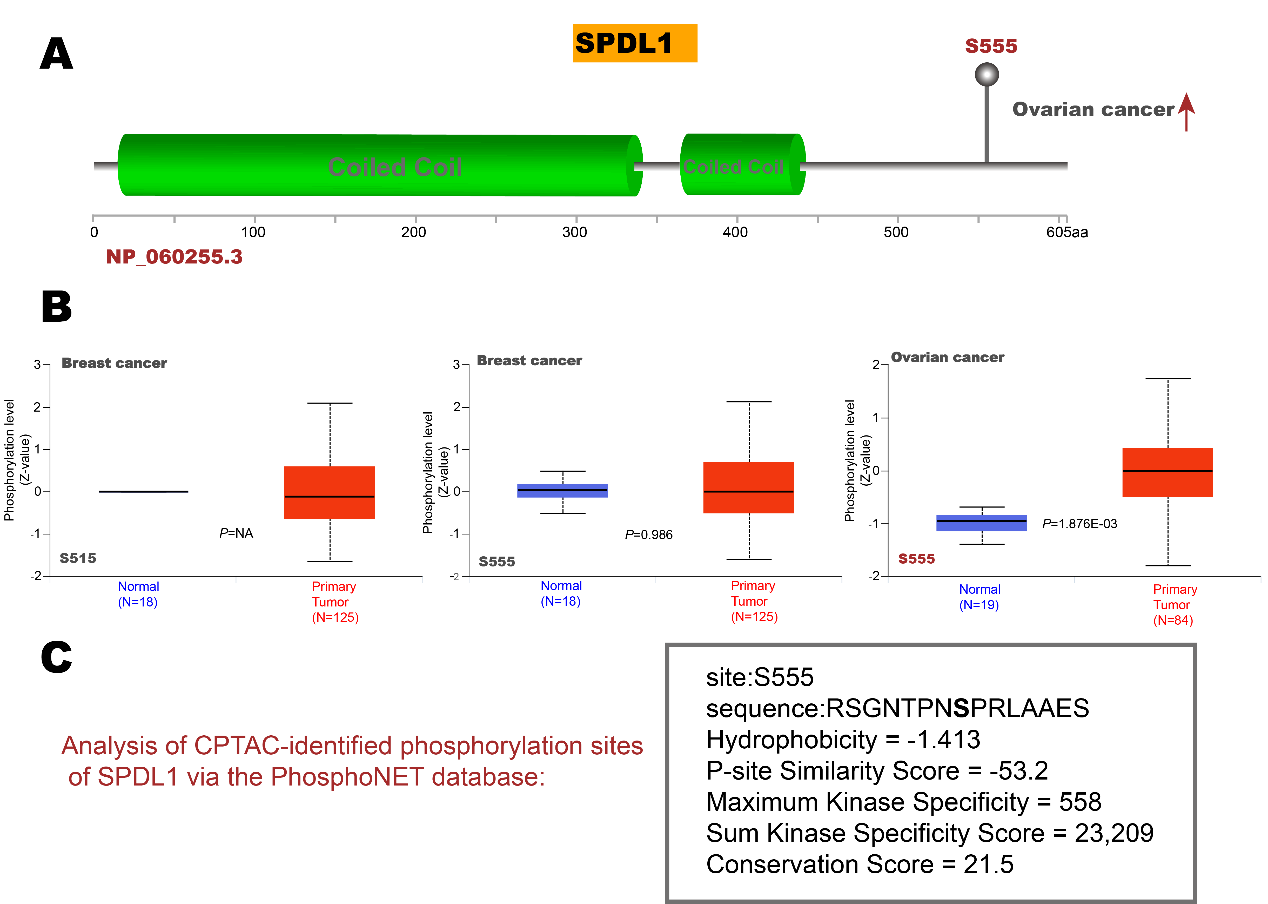


Fig. S9. Phosphorylation analysis of SPDL1 protein via the UALCAN. (A). The phosphoprotein site with significant differences. (B). The Expression level of SPDL1 phosphoprotein (NP_060255.3 site) between normal tissue and primary tissue of breast cancer and ovarian cancer in the box plots. (C). The detail information of S555 based on the PhosphoNET database.


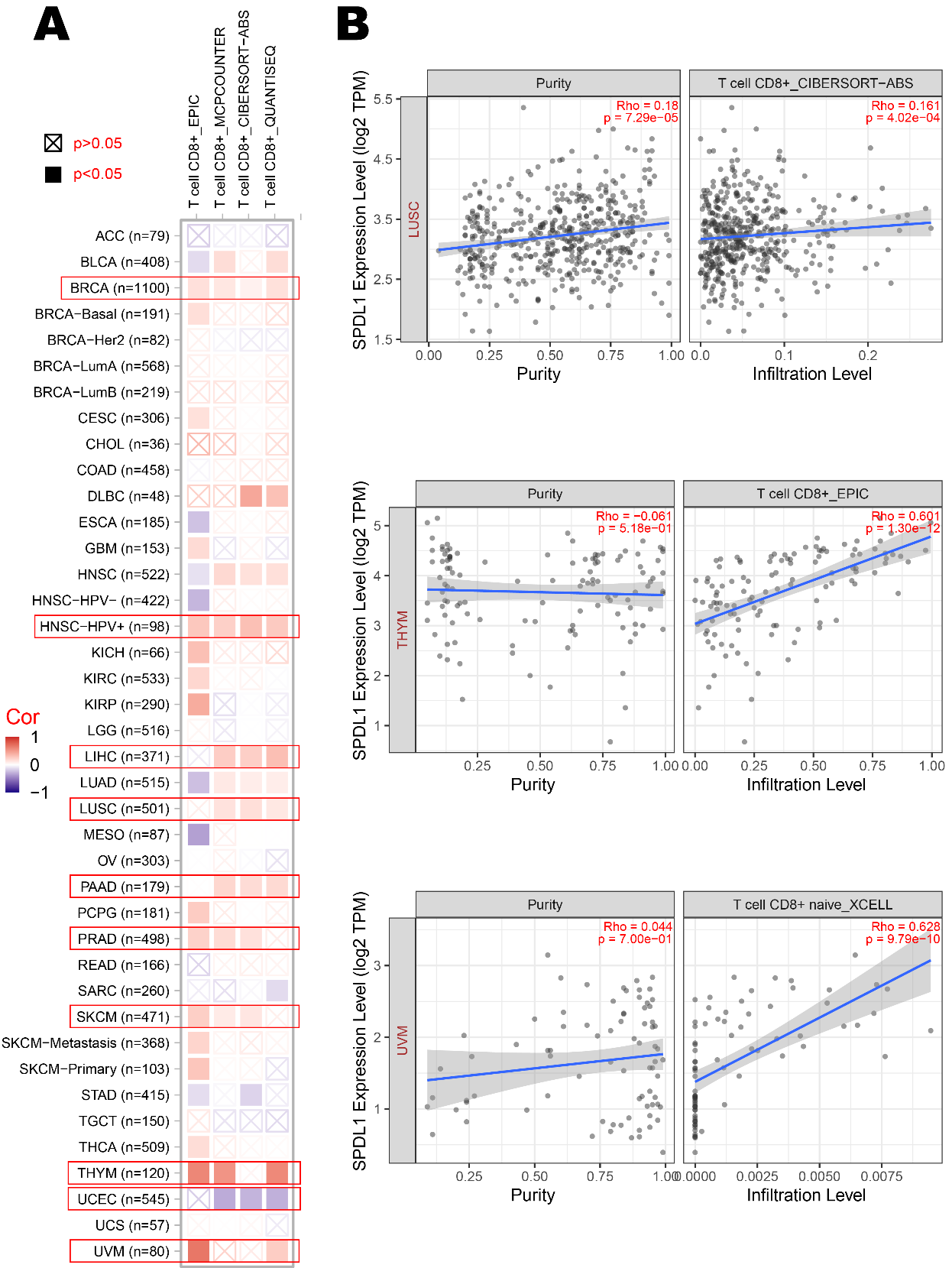


Fig. S10. Correlation between the SPDL1 gene expression and immune infiltration of CD8+ T-cells in all cancer types of TCGA. (A). Different algorithms (EPIC, MCPCOUNTER, CIBERSORT-ABS and QUANTISEQ) were used to explore the correlation between the infiltration level of CD8+ T-cells and the SPDL1 gene expression in all cancer types of TCGA. (B). The scatterplot data of the selected algorithm with the most statistically significant results.
