# Supplementary material for: Pan-cancer analysis combined with experiments explores the oncogenic role of spindle apparatus coiled-coil protein 1 (SPDL1)
Source: Cancer Cell Int. 2022 Jan 29;22:49. doi: 10.1186/s12935-022-02461-w (PMC8801078; doi:10.1186/s12935-022-02461-w)
Supplement: Supplementary file 3 — Additional file 3: Table S1. Subgroup analysis on the correlation of SPDL1 expression and prognosis of breast cancer cases. Table S2. Subgroup analysis on the correlation of SPDL1 expression and prognosis of lung cancer cases. Table S3. Subgroup analysis on the correlation of SPDL1 expression and prognosis of ovarian cancer cases. Table S4. Subgroup analysis on the correlation of SPDL1 expression and prognosis of gastric cancer cases. Table S5. Subgroup analysis on the correlation of SPDL1 expression and prognosis of liver cancer cases. [file 12935_2022_2461_MOESM3_ESM.docx]

**Table S1. Subgroup analysis on the correlation of *SPDL1* expression and prognosis of breast cancer cases.**

| **Factor** | **Subgroup** | **Sample size** | **OS** | | **RFS** | | **DMFS** | |
| --- | --- | --- | --- | --- | --- | --- | --- | --- |
|  |  |  | **HR** | ***P*** | **HR** | ***P*** | **HR** | ***P*** |
| **ER status** | ER positive | 3499 | 1.56 | 0.0061 | 1.47 | 3e-06 | 1.81 | 3.3e-05 |
|  | ER negative | 2168 | 0.67 | 0.027 | 1.2 | 0.073 | 1.16 | 0.25 |
| **TP53 status** | mutated | 272 | 0.61 | 0.15 | 1.33 | 0.24 | 1.65 | 0.21 |
|  | Wild type | 388 | 1.9 | 0.044 | 1.64 | 0.043 | 1.82 | 0.14 |
| **PR status** | PR positive | 1559 | 0.35 | 0.041 | 1.35 | 0.043 | 1.84 | 0.012 |
|  | PR negative | 1989 | 1.26 | 0.36 | 1.3 | 0.028 | 1.26 | 0.12 |
| **HER2 status** | HER2 positive | 1273 | 1.29 | 0.18 | 1.43 | 0.0012 | 1.39 | 0.059 |
|  | HER2 negative | 6262 | 1.43 | 0.0017 | 1.59 | 3.7e-15 | 1.62 | 2.9e-07 |
| **Grade** | Grade 1 | 576 | 3.5 | 0.0048 | 2.03 | 0.005 | 2.65 | 0.021 |
|  | Grade 2 | 1795 | 0.79 | 0.24 | 1.46 | 0.0015 | 1.52 | 0.0086 |
|  | Grade 3 | 2058 | 1.2 | 0.25 | 1.31 | 0.0083 | 1.43 | 0.012 |
| **Intrinsic subtype** | Basal | 1494 | 0.57 | 0.005 | 1.53 | 0.00036 | 0.75 | 0.071 |
|  | Luminal A | 3511 | 1.71 | 0.0021 | 1.43 | 7e-05 | 1.65 | 0.00021 |
|  | Luminal B | 2015 | 1.21 | 0.28 | 1.54 | 1.2e-06 | 1.5 | 0.0051 |
|  | HER2+ | 515 | 0.76 | 0.35 | 1.44 | 0.065 | 1.67 | 0.075 |
| **Lymph node status** | Lymph node positive | 2153 | 0.72 | 0.083 | 1.47 | 5.2e-05 | 1.53 | 0.00082 |
|  | Lymph node negative | 2829 | 1.62 | 0.013 | 1.54 | 2.5e-07 | 1.69 | 4.6e-05 |
| **Pietenpol subtype** | Basal-like 1 | 418 | 0.58 | 0.16 | 1.91 | 0.0027 | 1.58 | 0.11 |
|  | Basal-like 2 | 165 | 2.63 | 0.049 | 2.36 | 0.033 | 2.55 | 0.027 |
|  | immunomodulatory | 462 | 0.58 | 0.22 | 0.52 | 0.0043 | 0.45 | 0.0041 |
|  | Mesenchymal | 382 | 0.59 | 0.17 | 1.61 | 0.026 | 0.64 | 0.15 |
|  | Mesenchymal stem-like | 201 | 0.46 | 0.14 | 0.73 | 0.42 | 2.17 | 0.13 |
|  | Luminal androgen receptor | 413 | 0.55 | 0.055 | 1.44 | 0.094 | 1.39 | 0.28 |

HR, hazard ratio; OS, overall survival; RFS, relapse free survival; DMFS, distant metastasis free survival;

ER, Estrogen receptor; PR, Progesterone receptor; HER2, human epidermal growth factor receptor-2;

TP53, Tumor Protein P53; NA, not available data; *P* value less than 0.05 is shown in bold.

**Table S2. Subgroup analysis on the correlation of *SPDL1* expression and prognosis of lung cancer cases.**

| **Factor** | **Subgroup** | **Sample size** | **OS** | | **FP** | | **PPS** | |
| --- | --- | --- | --- | --- | --- | --- | --- | --- |
|  |  |  | **HR** | ***P*** | **HR** | ***P*** | ***HR*** | ***P*** |
| **Histology** | adenocarcinoma | 865 | 0.61 | 2e-05 | 0.75 | 0.077 | 0.51 | 0.0037 |
|  | squamous cell carcinoma | 675 | 1.27 | 0.062 | 2.49 | 0.00067 | 1.46 | 0.47 |
| **gender** | female | 817 | 1.4 | 0.017 | 1.43 | 0.028 | 0.7 | 0.067 |
|  | male | 1387 | 0.18 | 0.051 | 1.47 | 0.012 | 0.58 | 0.0048 |
| **smoking history** | exclude those never smoked | 970 | 1.31 | 0.033 | 1.53 | 0.0014 | 0.7 | 0.014 |
|  | only those never smoked | 247 | 0.43 | 0.0025 | 0.44 | 0.00058 | 0.47 | 0.018 |
| **stage** | stage I | 652 | 0.64 | 0.0012 | 0.53 | 0.0056 | 0.35 | 0.00045 |
|  | stage II | 320 | 0.59 | 0.006 | 1.57 | 0.094 | 0.28 | 0.0016 |
|  | stage III | 70 | 1.27 | 0.4 | NA | NA | NA | NA |
| **grade** | grade I | 202 | 1.47 | 0.035 | 1.37 | 0.16 | 0.74 | 0.24 |
|  | grade II | 310 | 1.32 | 0.11 | 1.63 | 0.021 | 1.37 | 0.25 |
|  | grade III | 77 | 2.26 | 0.022 | 1.48 | 0.34 | 0.44 | 0.16 |
| **AJCC stage t** | t1 | 475 | 1.71 | 0.00024 | 2.41 | 0.0028 | 1.72 | 0.11 |
|  | t2 | 686 | 1.49 | 0.00042 | 2.1 | 5.6e-06 | 0.64 | 0.011 |
|  | t3 | 99 | 1.52 | 0.16 | 0.63 | 0.36 | NA | NA |
|  | t4 | 48 | 1.85 | 0.067 | NA | NA | NA | NA |
| **AJCC stage n** | n0 | 863 | 1.54 | 2e-04 | 1.79 | 0.00032 | 0.64 | 0.043 |
|  | n1 | 296 | 1.71 | 0.00079 | 2.19 | 0.00072 | 0.55 | 0.033 |
|  | n2 | 113 | 1.66 | 0.015 | 1.96 | 0.053 | 0.42 | 0.032 |
| **AJCC stage m** | m0 | 818 | 1.88 | 01.4e-09 | 2.08 | 0.0046 | 1.3 | 0.4 |
| **surgery** | only surgical margins negative | 730 | 0.7 | 0.0098 | 0.69 | 0.0039 | 0.76 | 0.074 |
| **radiotherapy** | no | 276 | 1.38 | 0.095 | 1.41 | 0.075 | 1.55 | 0.058 |
|  | yes | 73 | 1.44 | 0.18 | 0.73 | 0.33 | 1.67 | 0.1 |
| **chemotherapy** | no | 317 | 1.36 | 0.082 | 1.51 | 0.042 | 1.78 | 0.023 |
|  | yes | 178 | 1.44 | 0.24 | 2.33 | 0.00014 | 0.52 | 0.0077 |

HR, hazard ratio; AJCC，American Joint Committee on Cancer; OS, overall survival; FP, first progression; PPS, post progression survival; NA, not available data; *P* value less than 0.05 is shown in bold.

**Table S3. Subgroup analysis on the correlation of *SPDL1* expression and prognosis of ovarian cancer cases.**

| **Factor** | **Subgroup** | **Sample size** | **OS** | | **PFS** | | **PPS** | |
| --- | --- | --- | --- | --- | --- | --- | --- | --- |
|  |  |  | **HR** | ***p*** | **HR** | ***p*** | **HR** | ***p*** |
| **Histology** | Endometrioid | 62 | 5.22 | 0.1 | 4.74 | 0.023 | NA | NA |
|  | Serous | 1232 | 1.16 | 0.082 | 1.23 | 0.0084 | 1.17 | 0.087 |
| **Stage** | Stage 1 | 107 | 4.72 | 0.028 | 10.24 | 0.0055 | NA | NA |
|  | Stage 2 | 72 | 2.08 | 0.17 | 2.27 | 0.033 | 2.35 | 0.15 |
|  | Stage 3 | 1079 | 1.26 | 0.0075 | 1.16 | 0.073 | 1.33 | 0.0051 |
|  | Stage 4 | 189 | 0.56 | 0.0033 | 1.76 | 0.0048 | 0.63 | 0.072 |
| **Grade** | Grade 1 | 56 | 1.43 | 0.46 | 0.45 | 0.14 | NA | NA |
|  | Grade 2 | 325 | 1.19 | 0.31 | 1.4 | 0.027 | 0.78 | 0.19 |
|  | Grade 3 | 1024 | 0.91 | 0.24 | 1.18 | 0.053 | 1.27 | 0.031 |
|  | Grade 4 | 21 | 3.77 | 0.02 | NA | NA | NA | NA |
| **TP53 mutation** | Mutated | 516 | 1.28 | 0.051 | 1.45 | 0.0025 | 1.23 | 0.14 |
|  | Wild type | 102 | 0.82 | 0.48 | 0.7 | 0.18 | 1.22 | 0.54 |
| **Debulk** | optimal | 802 | 1.17 | 0.15 | 1.31 | 0.0064 | 1.23 | 0.17 |
|  | suboptimal | 536 | 1.16 | 0.19 | 1.51 | 0.00048 | 1.3 | 0.074 |
| **Chemotherapy** | Contains platin | 1438 | 1.15 | 0.073 | 1.27 | 0.00035 | 1.19 | 0.064 |
|  | Contains Taxol | 821 | 1.13 | 0.19 | 1.21 | 0.029 | 1.12 | 0.27 |
|  | Contains Taxol+platin | 804 | 1.13 | 0.22 | 1.2 | 0.042 | 1.15 | 0.23 |
|  | Contains Avastin | 50 | 0.49 | 0.14 | 0.66 | 0.23 | 0.47 | 0.12 |
|  | Contains Docetaxel | 108 | 0.81 | 0.45 | 1.8 | 0.027 | 1.38 | 0.36 |
|  | Contains Gemcitabine | 135 | 0.72 | 0.13 | 1.52 | 0.038 | 0.82 | 0.44 |
|  | Contains Paclitaxel | 248 | 0.64 | 0.066 | 0.69 | 0.03 | 0.71 | 0.21 |
|  | Contains Topotecan | 119 | 0.71 | 0.11 | 1.43 | 0.086 | 0.68 | 0.098 |

HR, hazard ratio; OS, overall survival; PFS, progress free survival; PPS, post progression survival;

TP53, Tumor Protein P53; NA, not available data; *P* value less than 0.05 is shown in bold.

**Table S4. Subgroup analysis on the correlation of *SPDL1* expression and prognosis of gastric cancer cases.**

| **Factor** | **Subgroup** | **Sample size** | **OS** | | **FP** | | **PPS** | |
| --- | --- | --- | --- | --- | --- | --- | --- | --- |
|  |  |  | **HR** | ***P*** | **HR** | ***P*** | **HR** | ***P*** |
| **Gender** | Female | 244 | 0.42 | 7.2e-05 | 0.5 | 0.0038 | 0.26 | 2.6e-09 |
|  | Male | 567 | 0.77 | 0.018 | 1.15 | 0.31 | 0.73 | 0.025 |
| **Stage** | Stage 1 | 69 | 0.4 | 0.065 | 0.48 | 0.19 | 0 | 0.015 |
|  | Stage 2 | 145 | 0.37 | 0.0021 | 0.51 | 0.039 | 0.37 | 0.003 |
|  | Stage 3 | 319 | 0.6 | 0.0027 | 0.7 | 0.058 | 0.43 | 6.4e-05 |
|  | Stage 4 | 152 | 0.75 | 0.14 | 0.82 | 0.35 | 0.58 | 0.026 |
| **Stage t** | t2 | 253 | 0.62 | 0.029 | 0.7 | 0.1 | 0.46 | 0.00074 |
|  | t3 | 208 | 0.62 | 0.0098 | 0.72 | 0.074 | 0.46 | 0.00016 |
|  | t4 | 39 | 0.72 | 0.43 | 1.8 | 0.24 | 0.58 | 0.25 |
| **Stage n** | n0 | 76 | 0.3 | 0.014 | 0.33 | 0.022 | 0.17 | 0.00095 |
|  | n1 | 232 | 0.49 | 0.0011 | 0.59 | 0.014 | 0.42 | 0.00026 |
|  | n2 | 129 | 0.58 | 0.017 | 0.7 | 0.13 | 0.41 | 0.00032 |
|  | n3 | 76 | 0.46 | 0.0057 | 0.58 | 0.049 | 0.4 | 0.0026 |
| **Stage m** | m0 | 459 | 0.61 | 0.00052 | 0.71 | 0.014 | 0.4 | 1.4e-09 |
|  | m1 | 58 | 0.73 | 0.3 | 1.27 | 0.44 | 0.57 | 0.16 |
| **HER2** | negative | 641 | 0.62 | 3.3e-05 | 0.69 | 0.0053 | 0.49 | 1e-06 |
|  | positive | 425 | 1.52 | 0.0041 | 2.04 | 0.00049 | 0.67 | 0.05 |
| **Lauren classification** | Instestinal | 336 | 0.56 | 0.00071 | 0.74 | 0.1 | 0.44 | 0.00017 |
|  | Diffuse | 248 | 0.57 | 0.0015 | 0.62 | 0.0074 | 0.49 | 2e-04 |
|  | Mixed | 33 | 0.29 | 0.086 | 2.33 | 0.088 | NA | NA |
| **Differentiation** | Poorly | 166 | 1.71 | 0.028 | 1.67 | 0.031 | 2.17 | 0.029 |
|  | Moderately | 67 | 0.61 | 0.2 | 0.62 | 0.19 | 2.8 | 0.044 |
|  | Well | 32 | 3.23 | 0.05 | NA | NA | NA | NA |
| **Treatment** | Surgery alone | 393 | 0.62 | 0.0018 | 0.71 | 0.025 | 0.45 | 5.5E-07 |
|  | 5-Fu based adjuvant | 158 | 2.29 | 5.8e-05 | 2.02 | 0.00049 | 1.53 | 0.018 |
| **Perforation** | No | 169 | 1.43 | 0.077 | 1.42 | 0.074 | 1.72 | 0.067 |

HR, hazard ratio; OS, overall survival; FP, first progression; PPS, post progression survival;

HER2, human epidermal growth factor receptor-2; NA, not available data; *P* value less than 0.05 is shown in bold.

**Table S5. Subgroup analysis on the correlation of *SPDL1* expression and prognosis of liver cancer cases.**

| **Factor** | **Subgroup** | **Sample size** | **OS** | | **PFS** | | **RFS** | | **DSS** | |
| --- | --- | --- | --- | --- | --- | --- | --- | --- | --- | --- |
|  |  |  | **HR** | ***P*** | **HR** | ***P*** | **HR** | ***P*** | **HR** | ***P*** |
| **Stage** | Stage 1 | 171 | 2.02 | 0.024 | 2.08 | 0.02 | 1.73 | 0.052 | 2.32 | 0.059 |
|  | Stage 2 | 86 | 2.92 | 0.0052 | 2.42 | 0.0036 | 2.13 | 0.029 | 5.99 | 0.00071 |
|  | Stage 3 | 85 | 2.47 | 0.019 | 2.79 | 0.0031 | 2.9 | 0.0059 | 2.66 | 0.0065 |
| **Grade** | Grade 1 | 55 | 4.38 | 0.0019 | 2.57 | 0.016 | 0.62 | 0.32 | 4.01 | 0.021 |
|  | Grade 2 | 177 | 2.56 | 0.00039 | 3.52 | 1.2e-08 | 2.87 | 2.9e-05 | 4.37 | 3.4e-06 |
|  | Grade 3 | 122 | 2.8 | 0.00059 | 2 | 0.0066 | 1.98 | 0.014 | 3.07 | 0.0026 |
| **AJCC_T** | T1 | 181 | 2.1 | 0.013 | 1.9 | 0.0099 | 1.85 | 0.023 | 2.51 | 0.023 |
|  | T2 | 94 | 2.74 | 0.0048 | 2.63 | 0.00074 | 2.71 | 0.0027 | 5.91 | 0.00046 |
|  | T3 | 80 | 1.99 | 0.024 | 2.36 | 0.015 | 2.09 | 0.045 | 2.43 | 0.016 |
| **Gender** | Female | 121 | 3.26 | 8.8e-05 | 2.4 | 0.0039 | 2.27 | 0.0087 | 3.78 | 0.00075 |
|  | Male | 250 | 2.57 | 1.7e-05 | 2.25 | 9.2e-06 | 2.15 | 0.00017 | 4.23 | 1.3e-07 |
| **Vascular invasion** | None | 205 | 2.09 | 0.0052 | 2.11 | 0.0026 | 1.9 | 0.015 | 2.57 | 0.0087 |
|  | micro | 93 | 1.93 | 0.12 | 2.69 | 6e-04 | 2.55 | 0.005 | 2.37 | 0.11 |
| **Race** | White | 184 | 1.75 | 0.019 | 2.35 | 3.7e-05 | 2.06 | 0.0024 | 2.35 | 0.0066 |
|  | Asian | 158 | 5.06 | 1.3e-08 | 2.55 | 0.00012 | 2.59 | 0.00027 | 7.15 | 9.1e-08 |
| **Alcohol consumption** | Yes | 117 | 1.79 | 0.13 | 2.94 | 0.00016 | 2.55 | 0.0043 | 2 | 0.055 |
|  | none | 205 | 2.77 | 1.2e-05 | 2.51 | 1.8e-05 | 1.88 | 0.0085 | 4.74 | 1.7e-07 |
| **Hepatitis virus** | Yes | 153 | 2.15 | 0.02 | 1.63 | 0.045 | 1.4 | 0.19 | 3.57 | 0.0012 |
|  | none | 169 | 2.5 | 5.4e-05 | 3.82 | 3.2e-09 | 2.81 | 4.7e-05 | 3.68 | 2.8e-06 |

HR, hazard ratio; AJCC，American Joint Committee on Cancer; OS, overall survival; PFS, progress free survival;

RFS, relapse free survival; DSS, disease specific survival; NA, not available data; *P* value less than 0.05 is shown in bold.
